# Lipid‐Polymer Nanoparticles Mediate Compartmentalized Delivery of Cas9 and sgRNA for Glioblastoma Vasculature and Immune Reprogramming

**DOI:** 10.1002/advs.202309314

**Published:** 2024-06-23

**Authors:** Huaqing Zhang, Wenxin Jiang, Tingting Song, Mingjie Song, Shengyu Liu, Jianping Zhou, Hao Cheng, Yang Ding

**Affiliations:** ^1^ State Key Laboratory of Natural Medicines Department of Pharmaceutics China Pharmaceutical University Nanjing 210009 China; ^2^ State Key Laboratory of Functions and Applications of Medicinal Plants Guizhou Medical University Guiyang 550014 China

**Keywords:** Cas9 and sgRNA, compartmentalized delivery, glioblastoma therapy, lipid‐polymer nanoparticles, STAT3 gene editing

## Abstract

Hypervascularized glioblastoma is naturally sensitive to anti‐angiogenesis but suffers from low efficacy of transient vasculature normalization. In this study, a lipid‐polymer nanoparticle is synthesized to execute compartmentalized Cas9 and sgRNA delivery for a permanent vasculature editing strategy by knocking out the signal transducer and activator of transcription 3 (STAT3). The phenylboronic acid branched cationic polymer is designed to condense sgRNA electrostatically (inner compartment) and patch Cas9 coordinatively (outer compartment), followed by liposomal hybridization with angiopep‐2 decoration for blood–brain barrier (BBB) penetration. The lipid‐polymer nanoparticles can reach glioblastoma within 2 h post intravenous administration, and hypoxia in tumor cells triggers charge‐elimination and degradation of the cationic polymer for burst release of Cas9 and sgRNA, accompanied by instant Cas9 RNP assembly, yielding ≈50% STAT3 knockout. The downregulation of downstream vascular endothelial growth factor (VEGF) reprograms vasculature normalization to improve immune infiltration, collaborating with interleukin‐6 (IL‐6) and interleukin‐10 (IL‐10) reduction to develop anti‐glioblastoma responses. Collectively, the combinational assembly for compartmentalized Cas9/sgRNA delivery provides a potential solution in glioblastoma therapy.

## Graphical abstract

A lipid‐polymer hybrid nanoparticle is synthesized to perform compartmentalized Cas9 and sgRNA delivery for a permanent vasculature editing strategy by knocking out the signal transducer and activator of transcription 3 (STAT3). Downstream VEGF downregulation reprograms vasculature normalization to improve immune infiltration, collaborating with IL‐6 and IL‐10 reduction to develop anti‐glioblastoma responses.

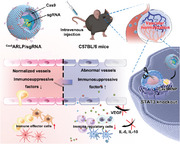

## 1 Introduction

Glioblastoma (GBM) is characterized by hypervascularization, and its proliferation, invasion, and migration seriously depend on angiogenesis, leading to natural sensitivity to antiangiogenic treatment.^[^ ^1^ ^]^ Multiple clinical trials on coadministration of antiangiogenic drugs and immune checkpoint inhibitors have revealed promising therapeutic benefits.^[^ ^2^ ^]^ The underlying mechanism is that vasculature normalization promotes immune cell infiltration and mitigates hypoxia.^[^ ^3^ ^]^ Clinical histopathological analysis of glioblastoma has identified signal transducer and activator of transcription 3 (STAT3) as the pivotal antiangiogenic and immune treatment target,^[^ ^4^ ^]^ due to the initial activation of various downstream effectors, such as the angiogenesis promoter vascular endothelial growth factor (VEGF) and the immunosuppressors interleukin‐6 (IL‐6) and interleukin‐10 (IL‐10).^[^ ^5^ ^]^ Great efforts have been devoted to the development of STAT3 inhibitors, such as CPA‐7 and WP1066, which have been applied in clinical studies for glioblastoma treatment with positive therapeutic effects.^[^ ^6^ ^]^ However, transient vasculature normalization and inducible resistance derived from reversible protein target deactivation critically hamper antitumor efficacy.^[^ ^7^ ^]^ As a result, long‐acting vasculature reprogramming by genomic editing has been identified as a promising strategy for glioblastoma therapy.

The clustered regularly interspaced short palindromic repeats (CRISPR)/associated protein 9 (CRISPR/Cas9) system is an efficient and versatile genome editing tool in biomedical applications.^[^ ^8^ ^]^ The site‐specific delivery of Cas9/sgRNA is the major obstacle in CRISPR/Cas9 genetic therapy because the immense differences between Cas9 and sgRNA in molecular weight and surface charge raise contradictory requirements for carriers.^[^ ^9^ ^]^ To solve this issue, Cas9 has been preincubated with sgRNA to generate anionic Cas9 ribonucleoprotein (Cas9 RNP), which was encapsulated by cationic carriers in existing studies.^[^ ^10^ ^]^ However, Cas9 RNP loading upon mono electrostatic interaction could hardly give high encapsulation efficiency due to the attenuated charge density and enormous molecular weight.^[^ ^11^ ^]^ To this end, we propose a phenylboronic acid‐installed quaternary ammonium polymeric structure with compartmentalized cargo loading capacity of electrostatic compression for nucleic acids and coordinative binding for proteins, which is supposed to satisfy the contradictory carrier requirements of Cas9 and sgRNA by combinational assembly.

For intracerebral drug delivery, high‐density lipoprotein (HDL) has emerged as an efficient blood–brain barrier (BBB) crossing carrier, deriving nanoparticles that mimic or intensify the merits of natural particulates.^[^ ^12^ ^]^ Inspired by the lipid‐protein biostructure, the apolipoprotein‐mimicking peptide of 4F (NH_2_‐FAEKFKEAVKDYFAKFWD‐COOH) could be designed for targeting peptide fusion and bionic decoration of lipid nanoparticles, which share both HDL‐like natural characteristics and site‐specific targeting capacity.^[^ ^13^ ^]^ Of note, the angiopep‐2 peptide (Ang) has been well‐organized in BBB‐crossing by low‐density lipoprotein receptor‐related protein‐1 (LRP‐1) meditated transcytosis, and the glioblastoma cell overexpressing LRP‐1 could appeal selective drug tumor accumulation.^[^ ^14^ ^]^ Moreover, Ang could be fused with the 4F peptide to modify lipid nanoparticles by anchoring them onto the lipid membrane.

**Scheme 1:**
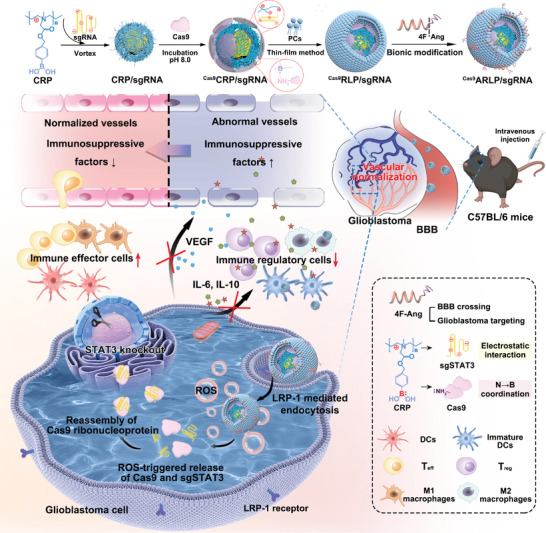
Schematic illustration of ^Cas9^ARLP/sgRNA fabrication and CRISPR/Cas9 genome editing‐mediated vasculature and immune reprogramming for glioblastoma therapy. The phenylboronic acid branched quaternary ammonium polymer (CRP) was synthesized to condense the sgRNA electrostatically (CRP/sgRNA) and patched the Cas9 coordinatively (^Cas9^CRP/sgRNA), followed by the construction of a 4F‐angiopep‐2 fusion peptide‐equipped liposome upon the nano complex to give the final lipid‐polymer hybrid nanoparticle of ^Cas9^ARLP/sgRNA. ^Cas9^ARLP/sgRNA nanoparticle treatment induced STAT3 knockout in glioblastoma. Downstream VEGF downregulation drives tumor vessel normalization to improve immune cell infiltration, collaborating with the reduction in immunosuppressive IL‐6 and IL‐10 to develop anti glioblastoma responses.

Herein, we designed lipid‐polymer hybrid nanoparticles by a combinational assembly strategy for compartmentalized Cas9‐ and STAT3‐targeting sgRNA (sgSTAT3) delivery to knock out STAT3 for tumor vessel normalization and immunostimulatory reprogramming in glioblastoma (**Scheme** **1**). For preparation, a cationic reactive oxygen species (ROS)‐responsive phenylboronic acid branched polymer (CRP) was synthesized to condense sgRNA electrostatically (inner compartment) and patch Cas9 coordinately (outer compartment). The generated nano‐complex was further hybridized with 4F‐angiopep‐2 fusion peptide‐decorated liposomes to give the final hybrid nanoparticles for BBB crossing, stabilization, and site‐specific accumulation. After intravenous administration, the hybrid nanoparticles mediated efficient BBB penetration and accumulated in the glioblastoma region via the LRP‐1 receptor. Thereafter, the concentrated ROS in tumor cells induced deboronation and charge elimination of the CRP polymer. The generated electroneutral zwitterionic polymer shared no interactions with either negatively charged sgRNA or positively charged Cas9, contributing to the rapid release of Cas9 and sgSTAT3. The Cas9 RNP was instantly assembled for STAT3 editing, which yielded ≈50% STAT3 knockout. STAT3 knockout blocked the secretion of VEGF, IL‐6, and IL‐10, causing an imbalance in the tumor ecosystem. The reprogrammed microenvironment drives tumor vessel normalization and further primes the immune system to develop anti‐glioblastoma responses. As designed, the hybrid nanoparticle‐mediated compartmentalized delivery of Cas9 and sgRNA could provide a potential strategy for genome‐editing glioblastoma therapy.

## 2 Results and Discussion

### 2.1 Preparation and Characterization of the Compartmentalized Nanoparticles

To construct the Cas9‐expressing plasmid (pCas9), pET‐28a was digested by the double enzymes EcoRI and HindIII (Figure S1A, Supporting Information), followed by ligation of the Cas9 gene with a 6×His tag fused at the C‐terminus of Cas9 (Figure S1B, Supporting Information). The obtained plasmid was transformed into *E. coli* DH5α for antibiotic resistance screening, and the subsequent amplification and purification of pCas9. The validated pCas9 was transformed into *E. coli* BL21(DE3) for Cas9 expression upon isopropyl 1‐thio‐β‐D‐galactopyranoside (IPTG) induction. The pCas9‐transformed bacteria were lysed and sampled for SDS‐PAGE analysis, and the results confirmed Cas9 expression with the maximum yield at 12 h of IPTG induction (Figure S2, Supporting Information). Cas9 was isolated by chromatography on nickel‐charged NTA‐agarose columns according to the manufacturer's directions (Figure S3, Supporting Information). The purity of the obtained Cas9 was 91.85% by reversed‐phase high‐performance liquid chromatography (RP‐HPLC) analysis (Figure S4A, Supporting Information), and the molecular weight of Cas9 was evaluated by using size exclusion chromatography high‐performance liquid chromatography (SEC‐HPLC), which was in accordance with the theoretically predicted molecular weight (Figure S4B, Supporting Information). Cas9 could completely bind with sgRNA at a Cas9/sgRNA ratio of 1, indicating a bioactive structure (Figure S5, Supporting Information). The gene cleavage capability of the purified Cas9 was further estimated with commercialized product (GenScript) as a positive control. The purified Cas9 shared cleavage efficiency of 20.58% and 48.20% against two DNA templates of EGFP, 3.96‐fold and 1.73‐fold higher than that of the GenScript product, which could promise gene editing activity in cells (Figure S6, Supporting Information).

The cationic ROS‐responsive phenylboronic acid (PBA) branched polymer (CRP) was synthesized by a three‐step approach (Figure S7, Supporting Information), and a nonresponsive PBA‐rich cationic polymer (PP) was designed as a control (Figure S8, Supporting Information). The chemical structure of the polymers was verified by ^1^H nuclear magnetic resonance (^1^H NMR), H–H correlation spectroscopy (H–H COSY), ^13^C‐NMR, and Fourier transform infrared spectroscopy (FTIR) spectra (Figures S9 and S10, Supporting Information). According to ^1^H NMR analysis, the single peak at δ 3.17 ppm was attributed to the introduced methyl group, compared with the polyethylenimine (PEI) spectra. In quaternized PEI (qua PEI), the single peak at δ 4.04 ppm was attributable to the (─CH_2_─) of the chloroacetic acid group. The chemical shift of the alkyl peak was increased due to the electron‐withdrawing effect from the generated quaternary ammonium. In the CRP polymer, the dual peaks at δ 7.38 and 7.68 ppm evidenced the conjugation of the PBA group. The peaks at δ 2.16–3.78 ppm were attributable to alkyl groups. For the PP polymer, the characteristic dual peaks at δ 8.13 and 8.19 ppm could be attributed to the amido‐linked PBA moieties, and the alkyl groups share a similar chemical shift to the CRP polymer due to the quaternary ammonium structure. In the H–H COSY spectra of the CRP polymer, obvious self‐coupling of aromatic hydrogens in PBA moieties and self‐coupling of alkyl hydrogens could be observed, but no H–H coupling between the benzene ring and alkyl groups was detected because of steric hindrance from the ester linkage (Figure S10A, Supporting Information). H–H COSY spectra of PP polymer revealed the correlations between hydrogen in the alkyl of quaternary ammonium and hydrogen in alkyl connected to amide, and self‐coupling of hydrogen in the benzene ring of PBA moieties (Figure S10B, Supporting Information). ^13^C‐NMR spectra were further employed to characterize the chemical structure of the CRP and PP polymers. In the CRP polymer, the dual peaks at δ 132.64 and 134.47 ppm were attributable to the benzene ring in the PBA group. The characteristic peak at δ 68.72 ppm was attributable to the ─CH_2_─ groups of chloroacetic acid and benzyl groups. The peaks at δ 40.77–50.09 ppm could be attributed to the alkyl carbon linked to quaternary ammonium (Figure S10C, Supporting Information). For PP polymer analysis, the dual peaks at δ 128.37 and 133.76 ppm were attributable to amide‐connected PBA groups. The peaks at δ 38.05–56.64 ppm could be attributed to the skeleton of the PEI polymer after quaternization (Figure S10D, Supporting Information). In the FTIR analysis, the characteristic absorption peaks of alkyl and aromatic nuclei were observed in both CRP and PP structures, while the carbonyl absorption peaks (≈1642.60, ≈1544.50 cm^−1^) of PP polymer further confirmed the existence of amide linkages. Moreover, the molecular weight of the polymers was estimated by size exclusion chromatography with multi‐angle laser‐light scattering and refractive index detection (SEC/MALLS/RI). Both CRP and PP polymers could give a single peak in SEC analysis, indicating the high purity of the polymers. The molecular weights of the CRP and PP polymers were calculated to be 51.77 and 59.66 kDa, respectively (Figure S11, Supporting Information).

**Figure 1:**
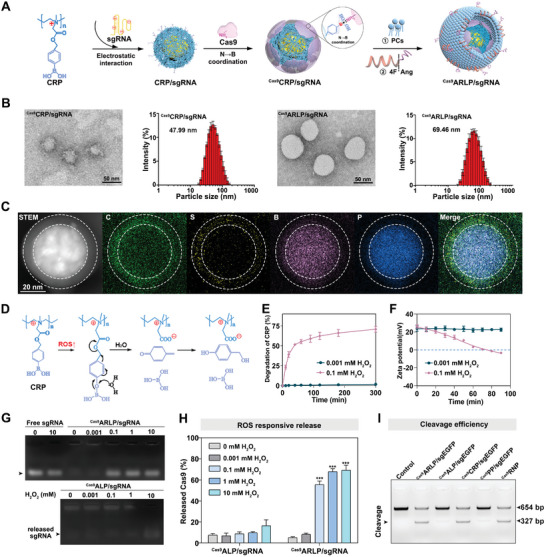
Fabrication and characterization of the hybrid ^Cas9^ARLP/sgRNA nanoparticle. A) Schematic illustration of hybrid ^Cas9^ARLP/sgRNA nanoparticle preparation. B) TEM image and diameter distribution of ^Cas9^CRP/sgRNA and ^Cas9^ARLP/sgRNA nanoparticles. C) STEM image and EDS elemental mapping of C, S, B, and P of ^Cas9^CRP/sgRNA nanoparticle. D) The mechanism of ROS‐triggered CRP degradation is accompanied by positive charge reversion. E) The degradation of CRP in H_2_O_2_ at various concentrations (*n* = 3). F) The ROS triggered the charge elimination of the CRP polymer (*n* = 3). ROS responsive release profile of sgRNA G) and Cas9 H) from ^Cas9^ARLP/sgRNA and nonresponsive ^Cas9^ALP/sgRNA made by the PP polymer when incubated with 0, 0.001, 0.1, 1, and 10 mm H_2_O_2_ at 37 °C for 12 h (*n* = 3). I) The in vitro cleavage efficiency after ^Cas9^ARLP/sgEGFP, ^Cas9^ALP/sgEGFP, ^Cas9^CRP/sgEGFP, ^Cas9^PP/sgEGFP, and Cas9 RNP incubated in 1 mm H_2_O_2_ at 37 °C for 12 h. Data are given as the means ± SD, ^***^ *p* < 0.001.

The CRP polymer shared the merits of electrostatic nucleic acid compression and coordinative protein binding, which could perfectly satisfy the contradictory demands of opposite charges from sgRNA and Cas9 on the carriers. Accordingly, the CRP polymer could assemble with sgRNA and Cas9 to construct the compartmentalized ^Cas9^CRP/sgRNA polyplex, with the sgRNA and Cas9 spatially separated loading at the core and surface of the nanoparticle. Compared with direct Cas9 RNP loading by the CRP polymer, the ^Cas9^CRP/sgRNA polyplex could give a nearly twofold encapsulation efficiency, accompanied by a small diameter and uniform distribution (Table S1, Supporting Information). The ^Cas9^CRP/sgRNA polyplex was further wrapped with the bionic peptide 4F‐Angiopep‐2 installed in the lipid membrane to give the final lipid‐polymer hybrid nanoparticles (^Cas9^ARLP/sgRNA) for biostability, BBB penetration, and tumor‐selective targeting (**Figure** **1**A). Gel electrophoresis demonstrated that CRP could effectively compress sgRNA at an N/P ratio of 6 (Figure S12, Supporting Information). X‐ray photoelectron spectroscopy (XPS) analysis revealed a binding energy peak split for boron when CRP interacted with Cas9, confirming coordinative protein binding (Figure S13, Supporting Information). To prepare the hybrid nanoparticles, sgRNA was compressed with CRP polymer to generate CRP/sgRNA nanocomplexes with a diameter of ≈40 nm (Figure S14, Table S2, Supporting Information). Thereafter, the Cas9 protein was coordinated onto the surface of CRP/sgRNA to obtain ^Cas9^CRP/sgRNA nanoparticles. The optimized ^Cas9^CRP_sgRNA_ also had a particle size of ≈48 nm, and the 4F‐Angiopep‐2‐installed lipid membrane‐wrapped ^Cas9^ARLP/sgRNA displayed a spheroid nanostructure with a diameter of ≈70 nm (Figure 1B). Slight size shrinkage was noticed in transmission electron microscopy (TEM) imaging compared with dynamic light scattering (DLS) data due to the loss of the hydration shell during sample preparation. The TEM overview validated the uniform diameter distribution of ^Cas9^CRP_sgRNA_ and ^Cas9^ARLP/sgRNA nanoparticles (Figure S15, Supporting Information). To further evidence compartmentalized loading of Cas9 and sgRNA, elemental mapping of ^Cas9^CRP/sgRNA nanoparticles was conducted via scanning transmission electron microscopy (STEM). The carbon (C) distribution represented whole nanoparticles, while boron (B) of CRP and phosphorus (P) of sgRNA were mainly located at the polymer core, and sulfur (S) of Cas9 was located at the outer sphere (Figure 1C). The results visually demonstrated that sgRNA and Cas9 were loaded separately in the nanoparticle. Moreover, the compartmentalized structure of ^Cas9^CRP/sgRNA nanoparticle was investigated by enzymic digestion. Upon incubation with RNase A and proteinase K, Cas9 on the outer shell of the ^Cas9^CRP/sgRNA nanoparticle could be digested, while the CRP‐compressed sgRNA in the core of the nanoparticle could remain intact. In direct Cas9 RNP loading nanoparticle of CRP/RNP, both Cas9 and sgRNA could maintain stability after the enzymic digestion, because CRP polymer wrapped the Cas9 RNP inside (Figure S16, Supporting Information). The results could further validate the compartmentalized loading of Cas9 and sgRNA. In addition, the dynamic binding behaviour between lipid‐polymer hybrid nanoparticles and 4F‐Angiopep‐2 was detected by surface plasmon resonance (SPR). 4F‐Angiopep‐2 could effectively bind with lipid‐polymer hybrid nanoparticles with a binding affinity of K_D_ = 1.58 µm (Figure S17, Supporting Information). Hence, a biomimetic modification mode of Angiopep‐2 was proposed for the preparation of ^Cas9^ARLP/sgRNA nanoparticles. During nanoparticle fabrication, zeta potential variations were monitored, where the positive zeta potential of Cas9, CRP/sgRNA, and ^Cas9^CRP/sgRNA nanoparticles was reversed after lipid membrane decoration. The negatively charged nanosurface was beneficial for the biostability of in vivo applications (Figure S18‐S19, Supporting Information). In comparison, naked ^Cas9^CRP/sgRNA nanoparticles shared a notable diameter increase when incubated in serum due to the redundant positive charges. Moreover, the integrity of sgRNA was essential for efficient gene editing performance. The sgRNA and CRP integrity in the ^Cas9^ARLP/sgRNA nanoparticles was estimated by gel electrophoresis and thin layer chromatography analysis, respectively (Figure S20, Supporting Information)

GBM cells share high oxidative stress due to metabolic demand and hypoxic conditions, where mitochondrial respiratory chain complexes harboring mitochondrial DNA mutations produce ROS in large quantities (≈0.1 mm).^[^ ^15^ ^]^ Accordingly, a ROS‐responsive CRP polymer was designed for high Cas9 and sgRNA loading and rapid release. The cationic CRP polymer could be oxidized by ROS and degraded into a zwitterionic polymer, phydroxybenzyl alcohol (HMP), and boric acid (Figure 1D). The electroneutral zwitterionic polymer had no interactions with either negatively charged sgRNA or positively charged Cas9, which could facilitate swift and complete release of Cas9 and sgRNA. Moreover, the transformation from a cationic to a zwitterionic polymer was supposed to minimize the underlying toxicity by charge elimination. CRP shared almost no disintegration under ROS levels in normal cells (0.001 mm), while a rapid degradation of 60% in 1 h was detected in 0.1 mm ROS medium, the equivalent ROS concentration in tumor cells (Figure 1E). The zeta potential of CRP in ROS medium was reduced from a positive charge to electric neutrality due to the generation of a zwitterionic polymer for complete payload release (Figure 1F). The responsive and synchronous release of Cas9 and sgRNA was investigated in a simulated oxidative environment. Upon ROS incubation, sgRNA could be released from ^Cas9^ARLP/sgRNA at ≈0.1 mm ROS, while no obvious sgRNA release was detected at the ROS concentration in normal cells (0.001 mm). The ROS‐controlled release manner could elevate the tumor selectivity of the gene editing. The nonresponsive ^Cas9^ALP/sgRNA nanoparticles hardly gave sgRNA release even at a much higher ROS concentration of 10 mm (Figure 1G). The results revealed that ROS triggered CRP degradation and that the subsequent positive charge annihilation was essential for rapid sgRNA release. Upon ROS‐triggered CRP degradation, the coordinative Cas9 binding was detached rapidly, and the cumulative Cas9 release reached 60% in 12 h (Figure 1H). Moreover, the carrier lacking Cas9 and sgRNA was subjected to cleavage evaluation, where no variation was detected in cleavage efficiency compared with the untreated Cas9 RNP (Figure 1I). The results suggested complete bioactivity retention by the spatially separated loading of Cas9 and sgRNA in ^Cas9^ARLP/sgRNA nanoparticles.

### 2.2 Blood–Brain Barrier Crossing Delivery of Cas9 and sgRNA to Glioblastoma

**Figure 2:**
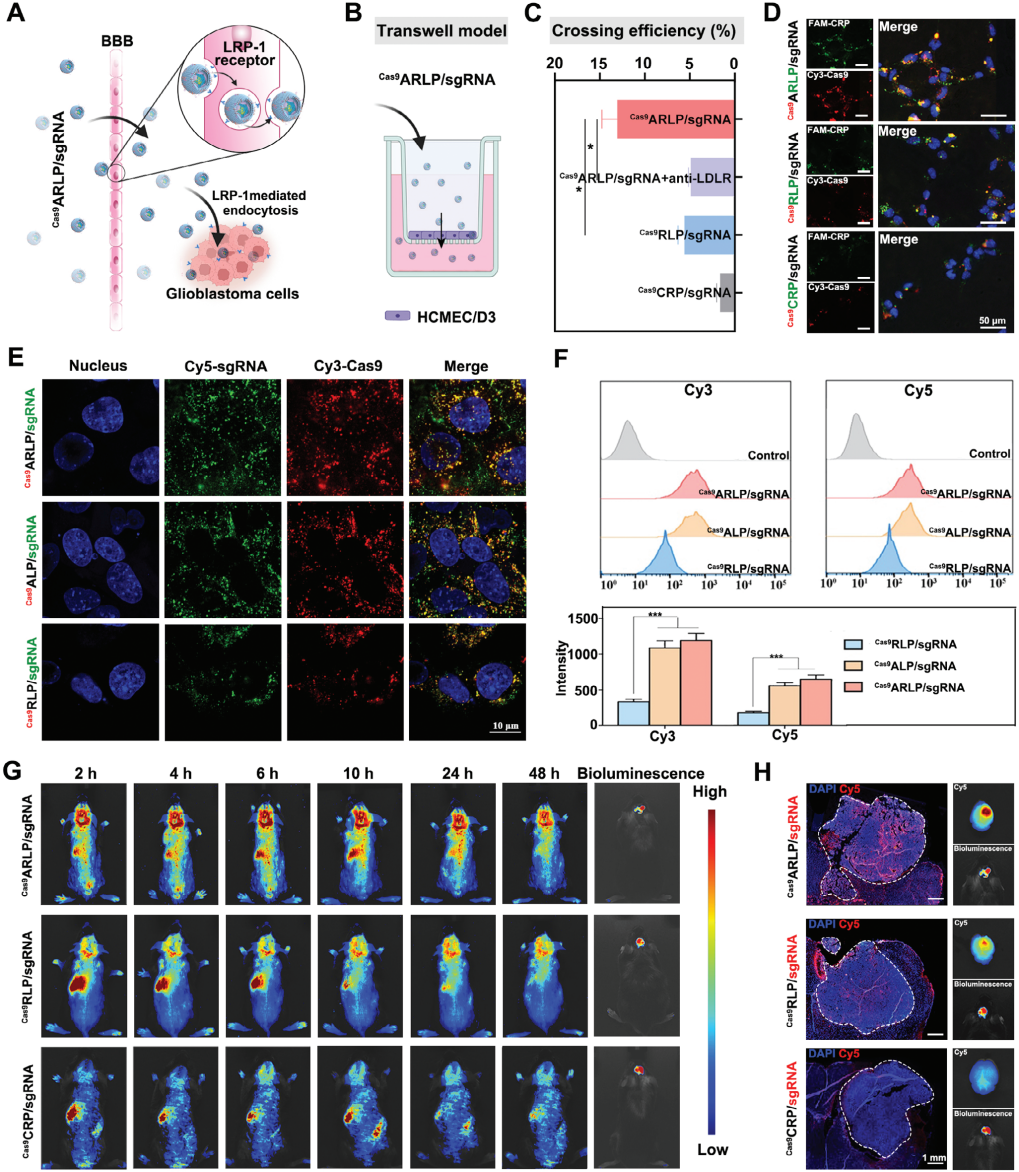
Blood–brain barrier (BBB) crossing delivery of Cas9 and sgRNA to glioblastoma. A) Schematic illustration of ^Cas9^ARLP/sgRNA nanoparticle‐mediated BBB crossing. B) Schematic illustration of the in vitro BBB model. C) BBB crossing efficiency of the ^Cas9^ARLP/sgRNA, ^Cas9^RLP/sgRNA, ^Cas9^CRP/sgRNA and ^Cas9^ARLP/sgRNA+anti‐LDLR groups (*n* = 3). HCMEC/D3 cells were pretreated with anti‐LDLR in the ^Cas9^ARLP/sgRNA+anti‐LDLR group to occupy LDLR before incubation with ^Cas9^ARLP/sgRNA nanoparticles. D) GL261 cell uptake of ^Cas9^ARLP/sgRNA, ^Cas9^RLP/sgRNA, and ^Cas9^CRP/sgRNA after crossing the hCMEC/D3‐BBB monolayer model. E) The intracellular distribution of Cas9 and sgRNA mediated by nanoparticles of ^Cas9^ARLP/sgRNA, ^Cas9^ALP/sgRNA, and ^Cas9^RLP/sgRNA. F) Flow cytometry analysis of Cas9 and sgRNA uptake mediated by ^Cas9^ARLP/sgRNA, ^Cas9^ALP/sgRNA, and ^Cas9^RLP/sgRNA nanoparticles (*n* = 3). G) In vivo biodistribution of nanoparticles imaged under the Cy5 channel. H) Ex vivo fluorescence images of brains and colocalization analysis of tumors with nanoparticles. Data are given as means ± SD, ^*^ *p* < 0.05, ^***^ *p* < 0.001.

The blood–brain barrier (BBB) hampers intracerebral drug shuttling and leads to limited therapeutic benefits in glioblastoma therapy. The nanoparticles of ^Cas9^ARLP/sgRNA could cross the BBB via recognition of low‐density lipoprotein receptor‐related protein‐1 (LRP‐1) (**Figure** **2**A). To evaluate the BBB‐penetrating efficiency of the obtained nanoparticles, an in vitro BBB model was established by human cerebral microvascular endothelial cell (hCMEC/D3) culture in the upper chamber of a Transwell (Figure 2B). Transendothelial electrical resistance (TEER) was monitored to evaluate the formation of a BBB monolayer with tight junctions. When TEER reached 100–120 Ω cm^−2^, hCMEC‐D3‐BBB model was ready for the BBB penetration efficiency investigation (Figure S21, Supporting Information). Fluorescence‐labeled nanoparticles were added to the upper chambers and cultured for 4 h. Thereafter, the fluorescence intensity of the culture medium in the lower chamber was estimated to calculate the BBB penetration efficiency. 4F‐Angiopep‐2‐modified ^Cas9^ARLP/sgRNA nanoparticles shared the highest BBB permeability ratio of ≈13% compared with the ^Cas9^RLP/sgRNA and ^Cas9^CRP/sgRNA groups. The BBB penetration ratio of ^Cas9^ARLP/sgRNA was sharply decreased when LDL receptor (LDLR) on BBB model cells was pre‐saturated by anti‐LDLR (Figure 2C). The results indicated that efficient BBB penetration of ^Cas9^ARLP/sgRNA nanoparticles was mainly mediated by LDLR‐facilitated transcytosis. The ^Cas9^ARLP/sgRNA nanoparticles could be internalized by mouse glioma 261 (GL261) cells with high efficiency, but the cellular uptake of ^Cas9^RLP/sgRNA nanoparticles was dramatically reduced, demonstrating the efficient endocytosis mediated by Angiopep‐2 decoration to bind LDLR overexpressed on tumor cells (Figure S22, Supporting Information). The nanoparticle‐containing medium collected from the lower chamber of the BBB penetration evaluation was further incubated with GL261 cells, and the cellular uptake was in accordance with that of the untreated nanoparticles (Figure 2D). The results demonstrated that ^Cas9^ARLP/sgRNA nanoparticles could maintain complete nanostructure after crossing the BBB to ensure efficient internalization by tumor cells.

To investigate the intracellular tracking and distribution of ^Cas9^ARLP/sgRNA in GL261 glioma cells, double‐labeled nanoparticles of ^Cy3‐Cas9^ARLP/Cy5‐sgRNA, ^Cy3‐Cas9^ALP/Cy5‐sgRNA and ^Cy3‐Cas9^RLP/Cy5‐sgRNA were prepared simultaneously to investigate the angiopep‐2 functional decoration and carrier ROS‐sensitivity capacity. As illustrated in Figure 2E,F, the intracellular fluorescence intensity of ^Cy3‐Cas9^ARLP/Cy5‐sgRNA and ^Cy3‐Cas9^ALP/Cy5‐sgRNA nanoparticles was significantly higher than that of the ^Cy3‐Cas9^RLP/Cy5‐sgRNA group due to angiopep‐2 decoration. No obvious difference was detected between the total fluorescence signals of the ^Cy3‐Cas9^ARLP/Cy5‐sgRNA and ^Cy3‐Cas9^ALP/Cy5‐sgRNA groups. Nevertheless, the overlapping fluorescence of Cas9 and sgRNA in the ROS‐responsive ^Cy3‐Cas9^ARLP/Cy5‐sgRNA group could be observed in the cell nucleus, while Cas9 and sgRNA in the ^Cy3‐Cas9^ALP/Cy5‐sgRNA group were mainly distributed in the cytoplasm. The results suggested that Cas9 and sgRNA were released rapidly from CRP nanoparticles upon the trigger of ROS in the cytoplasm and reassembled into Cas9 RNP, followed by nuclear translocation under the guidance of the nuclear localization sequence (NLS) fused in Cas9. The endonuclease delivery of Cas9 RNP could facilitate gene editing activity for effective oncotherapy. Moreover, to evaluate the biosafety of the lipid‐polymer nanoparticles, a non‐gene targeting sgRNA (sgNC) was used to fabricate the nanoparticle, and the sgNC nanoparticles shared no obvious toxicity toward GL261 cells, even at the high Cas9 concentration of 0.1 µg µL^−1^ (Figure S23, Supporting Information). The results indicated superior safety of the bioinspired nanoparticles, which was beneficial for in vivo applications.

In vivo, glioblastoma‐targeting capacity of nanoparticles was further evaluated with orthotopic GL261 glioma‐bearing C57 mice by utilizing non‐invasive near‐infrared optical imaging. Luciferase‐labeled GL261 cells (GL261‐Luc) were injected into the brain via a stereotaxic apparatus to establish an orthotopic glioma model. Bioluminescence could be used to easily monitor the glioma progression and indicate the glioma region. After intravenous administration, ^Cas9^ARLP/sgRNA nanoparticles displayed intense fluorescence in the brains of mice at 2 h; moreover, a strong fluorescent signal was detected and localized in the glioma regions at 48 h post‐administration, which was mainly due to the advanced biostability and tumor‐specific accumulation of nanoparticles (Figure 2G; Figure S24, Supporting Information). However, glioma targeting was significantly attenuated without Angiopep‐2 surface decoration due to insufficient BBB penetration and tumor cell selectivity. Moreover, ^Cas9^CRP/sgRNA nanoparticles gave the lowest cerebral fluorescence signal, which could be ascribed to the instability in circulation, as illustrated in Figure S17, Supporting Information. Ex vivo fluorescence images and corresponding ROI analysis (Figure 2H; Figures S25 and S26, Supporting Information) demonstrated the high intracranial distribution of ^Cas9^ARLP/sgRNA. The brain tissue from treated mice was collected and lysed for quantitative analysis of nanoparticle content in the brain, which was represented by sgRNA concentration. The sgRNA concentration of the ^Cas9^ARLP/sgRNA group in the brain was 2.3‐fold higher than that of the ^Cas9^RLP/sgRNA group, which confirmed that the nanoparticles crossed the BBB in vivo (Figure S27, Supporting Information). Furthermore, frozen brain slices witnessed the selective accumulation of ^Cas9^ARLP/sgRNA nanoparticles in the glioma region rather than distribution throughout the brain, indicating the efficient tumor‐homing capacity of the nanoparticles (Figure 2H). The tumor‐specific biodistribution could promise antitumor efficacy without causing severe toxicity to normal brain tissue.

### 2.3 In Vitro Vasculature and Immune Reprogramming by STAT3 Editing

**Figure 3:**
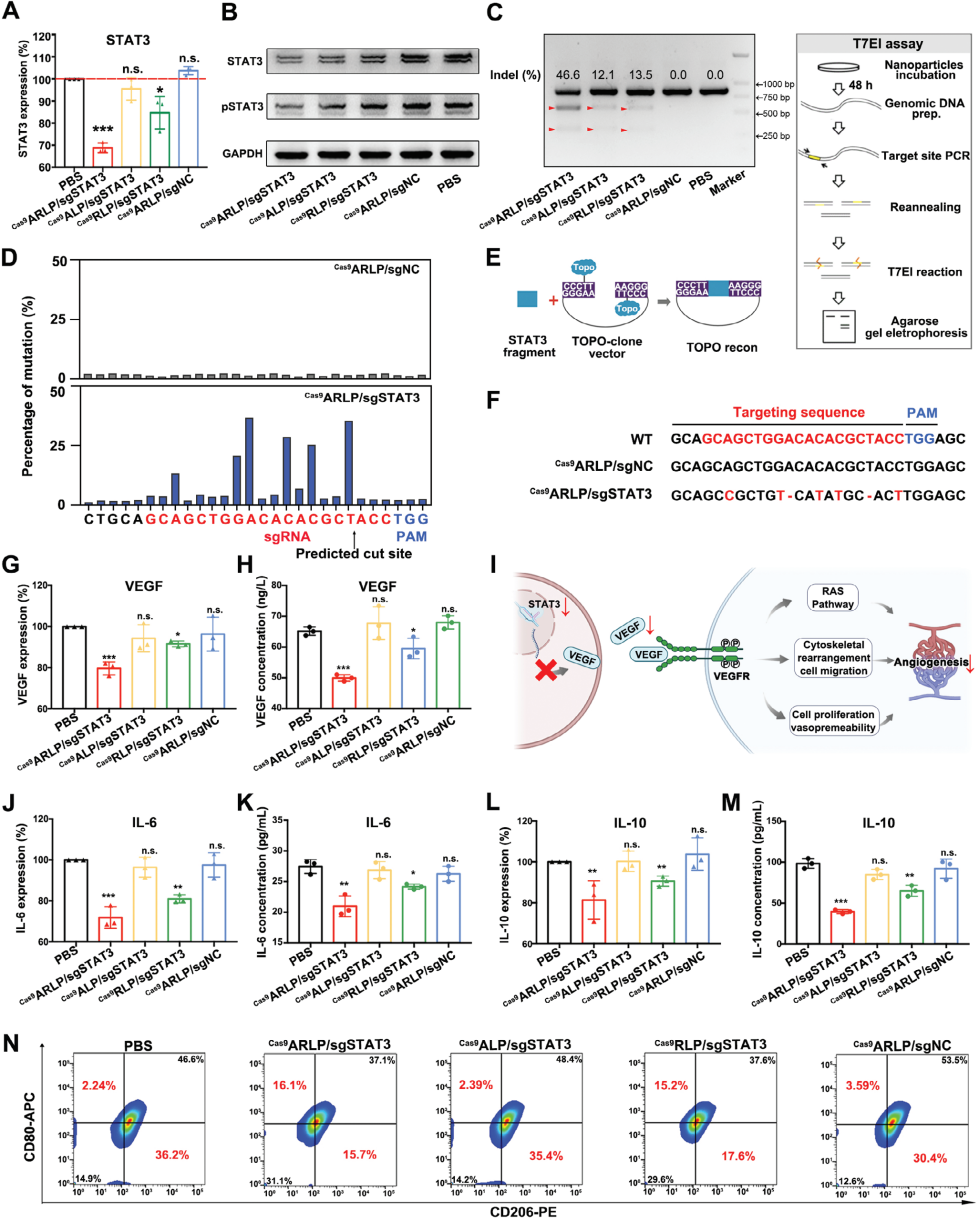
In vitro vasculature and immune reprogramming by compartmentalized Cas9 and sgRNA delivery mediated STAT3 editing. Quantitative real‐time PCR analysis (*n* = 3) A) and western blot analysis B) of STAT3 gene expression in GL261 cells after treatment with PBS, ^Cas9^ARLP/sgSTAT3, ^Cas9^ALP/sgSTAT3, ^Cas9^RLP/sgSTAT3 and ^Cas9^ARLP/sgNC. C) The indel efficiency of STAT3 in GL261 cells treated with the above preparations was determined by T7EI assay. D) Mutation spectra for reads with the proportion of each base in Sanger sequencing data are shown for every base proximal to the sgRNA target. E) Schematic of the TOPO cloning process. F) CRISPR edit analysis of Sanger sequencing data from GL261 cells after treatment. Quantitative real‐time PCR analysis G) and ELISA H) for VEGF expression after treatment (*n* = 3). I) Schematic illustration of VEGF downregulation‐induced vascular normalization. Quantitative real‐time PCR analysis J) and ELISA assay K) for VEGF expression after treatment with the preparations (*n* = 3). Quantitative real‐time PCR analysis L) and ELISA M) for VEGF expression after preparation treatment (*n* = 3). N) Proportion of M1/M2 polarization after M2 microglial cell culture in the supernatant of GL261 cells treated with PBS, ^Cas9^ARLP/sgSTAT3, ^Cas9^ALP/sgSTAT3, ^Cas9^RLP/sgSTAT3 and ^Cas9^ARLP/sgNC. Data are given as the means ± SD and significant differences are assessed by comparing PBS with other groups, ^*^ *p* < 0.05, ^**^ *p* < 0.01, ^***^ *p* < 0.001.

For STAT3 gene editing, we designed three STAT3 exon‐targeting sgRNA (sgSTAT3) sequences (Table S3, Supporting Information) and transfected them together with Cas9 into GL261 cells for evaluation of gene editing efficiency. The primers for gene expression analysis by qPCR are listed in Table S4 (Supporting Information). Among sgSTAT3s, sgSTAT3‐1 (5′‐GGTAGCGTGTGTCCAGCTGC‐3′) gave the highest STAT3 knockout efficiency (Figure S28, Supporting Information) and was selected for the following investigations, with a nongene targeting sgRNA (sgNC) sequence used as a negative control. As shown in **Figure** **3**A, STAT3 expression in the ^Cas9^ARLP/sgSTAT3 group was downregulated by 31.19% at the transcriptional level compared with that in the untreated group, which was significantly higher than that in the ^Cas9^ALP/sgSTAT3, ^Cas9^RLP/sgSTAT3, and ^Cas9^ARLP/sgNC groups. Western blot analysis gave similar results, where STAT3 and phosphorylated STAT3 (pSTAT3) were notably suppressed by ^Cas9^ARLP/sgSTAT3 (Figure 3B). In particular, pSTAT3 is an activated form with the capacity for transcription initiation, and its downregulation indicated direct inhibition of downstream genes. The results demonstrated that the high gene editing efficiency mainly resulted from ROS‐triggered controllable release of Cas9 and sgRNA. The ^Cas9^ARLP/sgSTAT3‐induced indels in the targeting locus were further detected by the T7E1 assay to estimate STAT3 knockout efficiency. Clear T7E1 cleavage bands were detected in the ^Cas9^ARLP/sgSTAT3‐treated group, with an indel rate of 46.62% (Figure 3C). The mutation spectra of the targeting locus were investigated by Sanger sequencing, and the results suggested a high mutation rate of approximately 30% in the region near the predicted cleavage site (Figure 3D). Therefore, the targeting locus of ^Cas9^ARLP/sgSTAT3‐mediated gene editing was analyzed by TOPO cloning and Sanger sequencing (Figure 3E). The sequencing results revealed 7 mutations of base deletion or substitution in the targeting locus (Figure 3F). To evaluate the off‐target risks, the potential off‐target sites were predicted by using Cas‐OFFinder (Table S5, Supporting Information), and the indel at the off‐target sites was detected by the T7E1 assay. There was no obvious indel band detected at the off‐target sites, indicating the negligible off‐target risks of ^Cas9^ARLP/sgSTAT3 treatment (Figure S29, Supporting Information).

As a point of convergence for numerous oncogenic signaling pathways, STAT3 regulates various downstream factors to support tumor development, where IL‐6, IL‐10, and vascular endothelial growth factor (VEGF) are the primary factors for immunosuppression and angiogenesis. VEGF expression in GL261 cells was significantly downregulated after ^Cas9^ARLP/sgSTAT3 transfection at both the transcriptional and translational levels (Figure 3G,H). VEGF downregulation inhibits neovascularization for vascular normalization, which benefits immune cell infiltration and hypoxia reversion (Figure 3I). The expression of tumor‐secreted IL‐6 and IL‐10 was further detected by qPCR and ELISA upon STAT3 knockout and indicated a notable reduction (Figure 3J–M). Immunosuppressive IL‐6 and IL‐10 could induce and maintain the M2‐like polarization of macrophages. To evaluate the impact of IL‐6 and IL‐10 downregulation on tumor‐promoting M2 macrophages, an M2 TAM model was established by IL‐4 treatment of macrophages. Thereafter, M2 macrophages were cultured with the medium of nanoparticle‐transfected GL‐261 cells, and the subpopulations of macrophages were detected by flow cytometry by utilizing CD206‐PE and CD80‐APC antibodies to label M2 and M1 subpopulations, respectively (Figure 3N). In the ^Cas9^ARLP/sgSTAT3 group, the M2 macrophage portion decreased to 43.37% of the PBS group, while the macrophage (M1) portion was 7.19‐fold higher than that in the PBS group. The results demonstrated that the ^Cas9^ARLP/sgSTAT3 design could reverse the immunosuppressive microenvironment through STAT3 knockout.

### 2.4 In Vivo Anti‐Glioblastoma Efficacy of the Compartmentalized Nanoparticle

**Figure 4:**
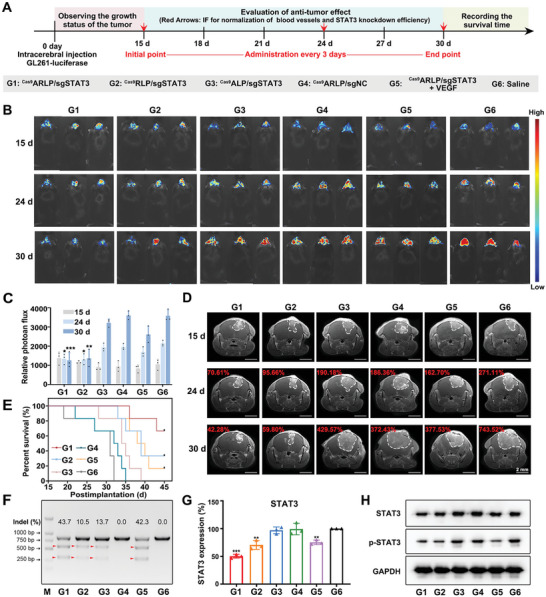
In vivo therapeutic efficacy of the compartmentalized nanoparticles. A) The experimental schedule of animal model establishment and mice treated with different preparations, including ^Cas9^ARLP/sgSTAT3, ^Cas9^RLP/sgSTAT3, ^Cas9^ALP/sgSTAT3, ^Cas9^ARLP/sgNC, ^Cas9^ARLP/sgSTAT3+VEGF and saline. MRI imaging was taken at 15 days, 24 days, and 30 days during the treatment, which is indicated by red arrows. The bioluminescence images B) and quantitative analysis C) of the intracranial GL261‐Luc glioblastoma mouse model at 15, 24, and 30 days after cell injection (*n* = 3). (D) MRI images of tumors after different treatments on the 15th, 24th, and 30th days. The percentage tumor volume variations were calculated. E) The survival time of different groups of intracranial glioblastoma‐bearing mice after various treatments (*n* = 6). F) The indel efficiency of STAT3 in glioblastoma treated with different preparations was determined by T7EI assay. The in vivo gene editing efficacy was evaluated by qPCR (*n* = 3) G) and western blot H). GAPDH was used as an internal reference. Data were given as means ± SD and significant differences were assessed by comparing G6 with other groups, ^*^ *p* < 0.05, ^**^ *p* < 0.01, ^***^ *p* < 0.001.

The therapeutic effects of ^Cas9^ARLP/sgSTAT3 nanoparticles in vivo were further investigated in an orthotopic GL261 glioma model. The glioma‐bearing mice were randomly divided into the following 6 groups: saline, ^Cas9^ARLP/sgSTAT3, ^Cas9^RLP/sgSTAT3, ^Cas9^ALP/sgSTAT3, ^Cas9^ARLP/sgNC, and ^Cas9^ARLP/sgSTAT3+VEGF. At 15 days post intracerebral injection of GL261‐Luc cells, the mice were administered with preparations every 3 days for a therapeutic regimen of 5 treatments (**Figure** **4**A). Glioma progression was monitored by MRI and bioluminescence imaging during the treatment period (Figure 4B–D). According to MRI analysis, the glioma section of the saline group increased by 7.44‐fold at the end of treatment compared to that at the first administration. The glioma region shrunk to 42.28% of the initial lesion after treatment with ^Cas9^ARLP/sgSTAT3 nanoparticles. The antitumor efficacy of ^Cas9^RLP/sgSTAT3 nanoparticles was attenuated with glioma region shrinkage to 59.80% due to a lack of BBB crossing and tumor‐specific affinity. In the unresponsive ^Cas9^ARLP/sgSTAT3 group, the glioma region increased by 4.29‐fold compared with the initial lesion. The results demonstrated that the rapid release of Cas9 and sgRNA was essential for antitumor efficacy. Since STAT3 knockout could simultaneously induce vascular normalization via VEGF downregulation and immunosuppression relief via IL‐6 and IL‐10 reduction, we implanted an intracranial needle to sustainably administer VEGF during treatment with ^Cas9^ARLP/sgSTAT3 and maintain normal VEGF levels in the glioblastoma region (Figure S30, Supporting Information). The ^Cas9^ARLP/sgSTAT3+VEGF group shared significantly fainter tumor‐suppression effects compared with the ^Cas9^ARLP/sgSTAT3 group. The results suggested that antitumor immune responses were seriously hampered by deficient immune infiltration from disordered vasculature. Therefore, vascular normalization is supposed to be a significant therapeutic mechanism in STAT3‐editing glioblastoma therapy. Bioluminescence imaging and quantitative analysis displayed similar results, where the ^Cas9^ARLP/sgSTAT3 group gave the highest tumor suppression. During the in vivo anti‐glioblastoma investigation, the mice were euthanized when they became moribund, i.e., fast weight loss of 20%, appetite loss, unstable to stand, listlessness and hypothermia, and the survival time was recorded. The survival curves demonstrated that treatment with ^Cas9^ARLP/sgSTAT3 extended the survival period of glioma‐bearing mice, with a survival rate of 60% at the end of the investigation. However, the mice in the unresponsive group of ^Cas9^ALP/sgSTAT3 all died in 41 days, further confirming the therapeutic benefit enhancement by ROS‐triggered site‐specific release of Cas9 and sgRNA (Figure 4E). Throughout the therapy, there was slight weight loss in ^Cas9^ARLP/sgSTAT3‐treated mice, indicating no obvious adverse effects (Figure S31, Supporting Information). To investigate the in vivo STAT3 knockout, the targeting gene sites from the glioblastoma were amplified for the T7EI assay. Clear cleavage bands were detected in the ^Cas9^ARLP/sgSTAT3‐treated mice, with an indel rate of 43.7% (Figure 4F). The in vivo gene editing efficiency was further estimated at both the transcriptional and translational levels (Figure 4G,H). The ^Cas9^ARLP/sgSTAT3 group revealed the supreme STAT3 editing of 49.62%, compared with other groups. The nanoparticles of ^Cas9^RLP/sgSTAT3 induced a lower STAT3 downregulation of 29.53%, mainly ascribed to the insufficient drug‐targeted distribution. Moreover, the STAT3 suppression capacity was attenuated in the ^Cas9^ARLP/sgSTAT3+VEGF group, probably due to the free VEGF initiating STAT3 activation. Western blot analysis showed that STAT3 and p‐STAT3 were simultaneously downregulated after ^Cas9^ARLP/sgSTAT3 treatment. In particular, the reduction in p‐STAT3, the activated form of STAT3, could effectively suppress the transcription of downstream genes, which could be attributed to the reversion of abnormal vascularization and immunosuppression.

### 2.5 In Vivo Vasculature and Immune Reprogramming by STAT3 Editing

**Figure 5:**
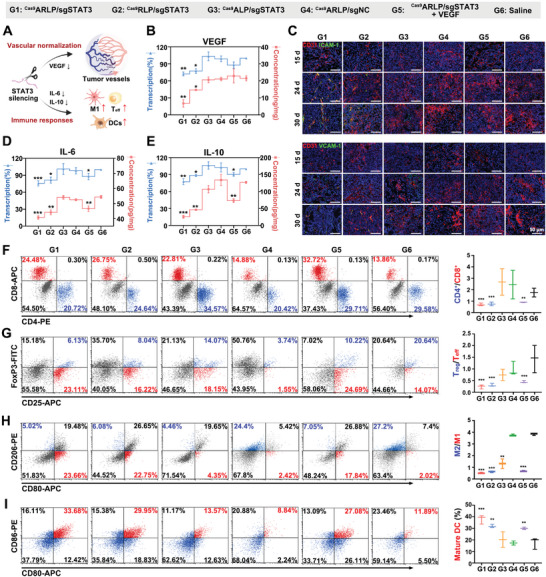
In vivo vascular normalization and immune activation by STAT3 editing. A) Schematic illustration of STAT3 knockout‐induced vascular normalization and tumor immune responses. B) qPCR analysis and ELISA assay for VEGF expression after treatment (*n* = 3). C) Immunofluorescence images of vascular normalization. CLSM images of colocalization of vessels in the tumor (red) and ICAM‐1 (green) or VCAM‐1 (green). qPCR analysis and ELISA for IL‐6 (D) and IL‐10 E) expression after preparation treatment (*n* = 3). F) Representative flow cytometry plots of CTL (CD3+, CD4−, CD8+, red) and T helper cells (CD3+, CD4+, CD8−, blue) in the brain, as well as the ratios of CD4+ to CD8+ cells (*n* = 3). G) Representative flow cytometry plots of T_eff_ cells (CD4+, CD25+, FoxP3−, red) and T_reg_ cells (CD4+, CD25+, FoxP3+, blue) in the brain, as well as the ratios of T_reg_ to T_eff_ cells (*n* = 3). H) Representative flow cytometry plots showing percentages of M1 macrophages (F4/80+, CD80+, CD206‐, red) and M2 macrophages (F4/80+, CD80‐, CD206+, blue), as well as the ratios of M2 to M1 macrophages (*n* = 3). I) Representative flow cytometry plots showing percentages of mature DCs (CD11c+, CD80+, CD86+, red) and all DCs (*n* = 3). Data are presented as mean ± SD and significant differences are assessed by comparing G6 with other groups, ^*^ *p* < 0.05, ^**^ *p* < 0.01, ^***^ *p* < 0.001.

^Cas9^ARLP/sgSTAT3 treatment also induced significant downregulation of IL‐10 and IL‐6 at both the transcriptional and translational levels (Figure 5D,E), which was supposed to collaborate with vascular normalization for immunosuppression reversion. As a result, the immune cells in glioma were extracted for subpopulation analysis of flow cytometry by biomarker labeling. As shown in Figure 5F, the ratio of T helper cells (Th) to cytotoxic T lymphocytes (CTLs) was decreased to 0.71 after ^Cas9^ARLP/sgSTAT3 treatment compared to 1.75 in the saline group. The increased CTL portion indicated the activation of tumor immune responses, which mediated tumor‐cell killing. Moreover, upon STAT3 editing therapy, the ratio of regulatory T cells (T_regs_) to effector T cells (T_effs_) was reduced by 83.3%, suggesting the effective alleviation of immune suppression (Figure 5G). In the tumor microenvironment, macrophages can be polarized into M2 macrophages, leading to immune suppression. The reduction in IL‐6 and IL‐10 levels by STAT3 editing was supposed to reverse polarization. The M2/M1 ratio revealed a remarkable decrease of 86.5% after ^Cas9^ARLP/sgSTAT3 administration (Figure 5H). Mature dendritic cells (DCs) are essential for antigen presentation but are hampered by the tumor microenvironment, leading to an increase in immature DCs. ^Cas9^ARLP/sgSTAT3 treatment resulted in a dramatic increase in DCs, which was 1.98‐fold higher than that in the saline group (Figure 5I). Notably, when vascular normalization was blocked by compensatory VEGF injection, ^Cas9^ARLP/sgSTAT3‐induced tumor immune responses were attenuated (Figure 5F–I), confirming the immune promotion of the normalized vessels via enhanced immune cell infiltration and oxygen infusion. The results validated that ^Cas9^ARLP/sgSTAT3 nanoparticles could reverse the immunosuppressive microenvironment in tumors and activate immune responses for tumor elimination.

STAT3 knockout could synchronously downregulate the angiogenesis regulator VEGF and the immunosuppression factors IL‐6 and IL‐10 for vascular normalization and antitumor immune activation. Normalized vessels facilitated tissue perfusion and effector T‐cell infiltration to further intensify antitumor efficiency (**Figure** **5**A). The intracranial implantation needle was employed for sustainable VEGF supplementation during the treatment to verify vascular normalization by VEGF downregulation and immunoreaction benefits from normalized vasculature. VEGF expression in tumors was detected by qPCR and ELISA, and ^Cas9^ARLP/sgSTAT3 induced remarkable VEGF reduction by 27.56% and 68.63% at the transcriptional and translational levels, respectively (Figure 5B). Moreover, VEGF transcription in the ^Cas9^ARLP/sgSTAT3+VEGF group was downregulated by ≈13%, while VEGF protein expression showed negligible variation compared with that in the saline group. The results demonstrated that the reduced VEGF protein by STAT3 editing was compensated through brain stereotaxic microinjection of VEGF. During treatment, tumor vessel progression was further investigated by immunofluorescence, with CD31 to indicate microvessel density and vascular cell adhesion molecule‐1 (VCAM‐1) and intercellular adhesion molecule‐1 (ICAM‐1) to distinguish normalized vessels (Figure 5C). Prior to the administration of preparations, no VCAM‐1‐ or ICAM‐1‐positive vessels could be observed in any of the tumor tissues. In the ^Cas9^ARLP/sgSTAT3‐treated group, the expression of VCAM‐1 and ICAM‐1 was significantly elevated as STAT3 editing therapy continued, with no vessel density increase. However, the ^Cas9^ARLP/sgSTAT3+VEGF group exhibited no VCAM‐1 or ICAM‐1 expression and a sharp increase in vessel density, which was similar to that of the saline group. Compensatory VEGF administration to block vascular normalization confirmed the mediation of the VEGF pathway after ^Cas9^ARLP/sgSTAT3 treatment. The normalized vessels in tumors could benefit deep tumor drug shuttling and immune infiltration.

For safety, hemolysis and potential in vivo adverse effects were further evaluated systematically. The hybrid bio‐nanoparticles indicated a negligible hemolytic rate below 2% at all test concentrations (Figure S32, Supporting Information), demonstrating safety and appropriateness for intravenous administration. Moreover, there was no significant difference between the groups of preparations and saline in biochemical parameters for evaluating the functions of the liver and kidney (Figure S33, Supporting Information). No obvious pathological abnormalities were observed in major organs in nanoparticle‐treated mice (Figure S34, Supporting Information). Collectively, the results confirmed that hybrid bio‐nanoparticles were safe and suitable for potential clinical applications.

## 3 Conclusion

In the present study, we developed novel hybrid nanoparticles of CRP polymer and 4F‐angiopep‐2 fusion peptide‐modified liposomes that enable efficient intracerebral Cas9 and sgRNA delivery for glioblastoma therapy. The CRP polymer provides the capacity of both gene compression and protein binding via electrostatic interaction and coordination, respectively, which enables the compartmentalized loading of Cas9 and sgRNA for optimized encapsulation. The bionic anchor of the angiopep‐2 peptide could direct nanoparticles to cross barriers of the BBB for glioblastoma‐specific accumulation. After internalization, the intracellular concentrated ROS in tumor cells could trigger the instant transformation of the cationic CRP polymer to a neutral zwitterionic polymer for swift Cas9 and sgSTAT3 release, accompanied by Cas9 RNP reassembly to knock out the STAT3 gene in the genome. Efficient STAT3 gene editing downregulated VEGF, IL‐6, and IL‐10 to reprogram disordered vasculature and the immunosuppressive microenvironment for glioblastoma therapy. The study provided pioneering compartmentalized delivery of CRISPR/Cas9 components for elevated genome editing. The bioinspired lipid‐peptide surface design represents a novel strategy for BBB penetration and tumor‐targeted drug shuttling. Collectively, the introduction of hybrid nanoparticles mediated site‐specific STAT3 gene knockout for advanced antiangiogenic and immune glioblastoma therapy.

## Conflict of Interest

The authors declare no conflict of interest.

## Supporting information

Supporting Information

## Data Availability

The data that support the findings of this study are available from the corresponding author upon reasonable request.
