# Supplementary material for: Lipid‐Polymer Nanoparticles Mediate Compartmentalized Delivery of Cas9 and sgRNA for Glioblastoma Vasculature and Immune Reprogramming
Source: Adv Sci (Weinh). 2024 Jun 23;11(32):2309314. doi: 10.1002/advs.202309314 (PMC11348121; doi:10.1002/advs.202309314)
Supplement: Supplementary file 1 — Supporting Information [file ADVS-11-2309314-s001.docx]

**Supporting Information**

**Lipid-Polymer Nanoparticles Mediate Compartmentalized Delivery of Cas9 and sgRNA for Glioblastoma Vasculature and Immune Reprogramming**

*Huaqing Zhang, Wenxin Jiang, Tingting Song, Mingjie Song, Shengyu Liu, Jianping Zhou*, Hao Cheng*, Yang Ding**

H. Zhang, W. Jiang, T. Song, M. Song, S. Liu, J. Zhou, H. Cheng, Y. Ding

State Key Laboratory of Natural Medicines, Department of Pharmaceutics, China Pharmaceutical University, Nanjing 210009, China

E-mail: dydszyzf@163.com; chenghao@cpu.edu.cn; zhoujianping@cpu.edu.cn

Y. Ding

State Key Laboratory of Functions and Applications of Medicinal Plants, Guizhou Medical University, Guiyang 550014, China

**Experimental Section**

**Materials and reagents.** Recombinant plasmids pET-28a (+) were purchased from General Biology Co., Ltd. Cas9-SV40 stranded DNA, commercial Cas9 protein and sgRNA were obtained from Genescript Biotechnology Co., Ltd. EcoRI, HindIII Restriction endonucleases, CutSmart buffer were purchased from New England Biolabs Biotechnology Co., Ltd. Kanamycin and isopropyl-β-d-thiogalactoside (IPTG) and 3-(4,5-Dimethylthiazol-2-yl)-2,5-diphenyltetrazolium bromide (MTT) were purchased from Yifeixue BioTech. T4 DNA Ligase and T4 DNA Reaction Buffer were purchased from Takara Bio Co., Ltd. 4F-Angiopep-2 peptide (NH_2_-FAEKFKEAVKDYFAKFWDGSGTFFYGGSRGKRNNFKTEEY-COOH) was purchased from Guoping Pharmaceutical Co., Ltd. (Anhui, China). Branched polyethyleneimine (PEI, 10 kDa), N-Hydroxy succinimide (NHS), paraformaldehyde, hematoxylin, eosin and hydrogen peroxide (H_2_O_2_) solution 30% (w/w) in H_2_O were purchased from Shanghai Aladdin Reagent Co., Ltd. Methyl iodide, 4-(bromomethyl) phenylboronic acid and 4-carboxyphenylboronic acid were provided by Shanghai Macklin Biochemical Technology Co., Ltd. Dialysis bag (MWCO: 3500 Da) was purchased from Shanghai Greenbird Co., Ltd. Phosphatidylcholine (95%) was purchased from Shanghai Taiwei Pharmaceutical Co., Ltd. Agarose was purchased from Shanghai Lianshuo biological Co., Ltd. RNA electrophoresis loading buffer was purchased from Shanghai Kanglang Biological Technology Co., Ltd. BCA protein detection kit, RIPA lysis buffer, phenylmethylsulfonyl fluoride (PMSF) solution were purchased from Beyotime Biotechnology Co., Ltd. Penicillin and streptomycin were purchased from Boc Sciences (USA). 5(6)-carboxyl luciferin succinimide ester (FAM-SE) was purchased from Shanghai Aladdin Reagent Co., Ltd. Live-dead cell staining kit was purchased from Thermo Fisher Scientific Co., Ltd. Annexin V-FITC/PI apoptosis detection kit was purchased from Shanghai Haoran Biotechnology Co., Ltd. Matrigel and transwell chambers were purchased from Becton, Dickinson and Company (USA). D-luciferin sodium salt was bought from FanBo Biochemistry Co., Ltd. *E. coli* BL21(DE3) competent cells were purchased from Tsingke Biotechnology Co.,Ltd.

**Cells and animals.** The mouse glioma cell line GL261, Luciferase-labeled GL261 cells, BV-2 microglia cells and brain endothelial hCMEC/D3 cells were from the Cell Bank of the Chinese Academy of Sciences (Shanghai, China). The cells were cultured in DMEM with 10% FBS at 37 ℃ in a humidified atmosphere with 5% CO_2_. Six-week-old male C57BL/6J mice were purchased from Qinglongshan Animal Center (Nanjing, Jiangsu, China). The mice were housed and maintained in a 12-hour light/dark regular environment. All animal experiments were carried out in accordance with the National Institute of Health Guide for the Care and Use of Laboratory Animals and approved by the China Pharmaceutical University Ethics Committee (2023-06-004).

**Purification and activity investigation of Cas9 protein.** The sequence of Cas9 was constructed into a pET-28a(+) plasmid vector via double enzyme digestion with EcoRI and HindIII, and ligation with T4 Ligase. The obtained Cas9-pET-28a(+) plasmid was transfected into *E. coli* BL21(DE3) and the recombinant Cas9 protein was expressed upon induction of isopropyl-β-d-thiogalactoside (IPTG). To detect the prokaryotic expression of Cas9, Escherichia coli was collected by centrifugation and lysed with NP40 solution upon sonication. After centrifugation, the supernatant liquid was sampled for SDS-PAGE analysis. Escherichia coli without IPTG induction was used as control. The prokaryoticly expressed Cas9 protein was purified by affinity chromatography with His-Trap HP nickel column. The eluent at various imidazole concentrations was subjected to SPS-PAGE to detect the Cas9 content. The Cas9 protein purity was determined by reversed-phase-high performance liquid chromatography (RP-HPLC) with Sepax GP-C18 column and size exclusion chromatography-high performance liquid chromatography (SEC-HPLC) at absorbance of 214 nm. The binding ability of purified Cas9 protein and sgRNA was investigated at various Cas9: sgRNA molar ratios by agarose gel electrophoresis retardation assay.

**Synthesis and characterization of polymers.** Synthesis and characterization of ROS responsive phenylboronic acid-rich cationic polymer (CRP). Tertiary amination product of PEI (tert-PEI) was synthesized by Eschweiler–Clarke reaction as previously reported^[1]^. Briefly, 0.269 g PEI was dissolved in 0.5 mL H_2_O and slowly added to 1 mL formic acid in ice bath, followed by addition of 1 mL formaldehyde solution (40%). The reactant was heated up to 90 ℃ and maintained for 8 h in oil bath under reflux condensation. The mixture was cooled to room temperature, followed by addition of 3.1 mL HCl (4 M) solution. The solvent was removed in vacuo and the residue was dissolved in 2.5 mL water. Thereafter, 1.6 mL NaOH (18 M) solution was added and mixed well. After extraction with 2.5 mL benzene for three times, the organic layer was dried by sodium carbonate and the tert-PEI was collected by vacuum evaporation. The tert-PEI was dissolved into DMF, and added into sodium chloroacetic acid solution (pH 9~10) for 6 h and dialysis in ultrapure water for 48 h, and then the qua-PEI was obtained by lyophilization. The qua-PEI and 4-(bromomethyl) phenylboronic acid were co-dissolved in the solvent (DMF: H_2_O = 6: 4). A few KI was added and stirred in dark for 24 h. After dialysis for 48 h, the CRP polymer was harvested by lyophilization. The chemical structure of CRP was verified by ^1^H-NMR, H-H correlation spectroscopy (H-H COSY), ^13^C-NMRand Fourier Transform Infrared Spectroscopy (FTIR).

Synthesis and characterization of unresponsive phenylboronic acid rich cationic polymer (PP). To explore the benefits of ROS triggered positive charge annihilation materials in drug, a nonresponsive phenylboronic acid-rich quaternized polymer (PP) was prepared as control. 55.3 mg 4-carboxyphenylboric acid was dissolved in 2 mL H_2_O, followed by addition of 127.8 mg EDC and 58.5 mg NHS. The mixture was stirred vigorously in ice-water bath for 1 h, after which 22.0 mg PEI dissolved in 1 mL DMF was added dropwise. After stirring at room temperature for 24 h, the reactant was dialyzed in ultrapure water for 24 h. The product was obtained by lyophilization and then dissolved in 2 mL DMF. 0.5 mL of methyl iodide was added to the mixture, followed by reaction in dark for 48 h and dialysis in ultrapure water for 48 h. The PP polymer was obtained by lyophilization, with its chemical structure verified by ^1^H-NMR, H-H COSY, ^13^C-NMR and FTIR.

Molecular weight of CRP and PP polymers was estimated by SEC/MALLS/RI. The SEC analysis was performed on a Waters 1515 instrument equipped Waters 4.6×30 mm guard column and three Waters WAT054466, WAT044226, WAT044223 columns (Polymer Laboratories: linear range of molecular weight = 500 - 4×10^6^ Da), and light scattering of HELEOS8+ (WYATT technology, USA) and differential refractive index detector using DMF (HPLC grade, containing 50 mmol/L LiBr) as the eluent at 35 °C with a flow rate of 1.0 mL/min.

The sgRNA compression and coordinative Cas9 conjugation capacity of CRP. The sgRNA was mixed with CRP polymer under vortex at various N/P molecular ratios. The generated CRP/sgRNA polyplexes were sampled for agarose gel electrophoresis analysis to detect the sgRNA compression. The Cas9 protein was incubate with CRP/sgRNA polyplexes at pH 7.5~8.0 for 6 h. The obtained ^Cas9^CRP/sgRNA solution was lyophilized to yield ^Cas9^CRP/sgRNA powder for X-ray photoelectron spectroscopy (XPS) analysis, with lyophilized CRP/sgRNA used as control.

ROS responsiveness of CRP polymer. To explore the ROS-triggered degradation of CRP polymer, the degradation product 4-hydroxymethyl-phenol was monitored in real time. The CRP polymer was dissolved in H_2_O_2_ solution (0.001 and 0.1 mM) and the solution was sampled at the predetermined time intervals for HPLC analysis (Lichrospher™ C18 column, 5 µm particle size, 250 mm × 4.6 mm) to detect 4-hydroxymethyl-phenol concentration at 220 nm wavelength, using 10% methanol aqueous solution as eluant at a flow velocity of 1.0 mL/min. Pure 4-hydroxymethyl-phenol was used as a standard. Moreover, at predetermined time intervals, the CRP solution was sampled to detect the zeta potential variations of CRP polymer upon oxidation.

**Preparation and characterization of ^Cas9^CRP/sgRNA.** The CRP/sgRNA complex was prepared by vortex at a N/P molar ratio of 6. CRP solution in water (10 mg mL^-1^) was swirled with sgRNA solution in water (100 μg mL^-1^) at a ratio of 1: 5 (v/v). Free CRP polymer and sgRNA were removed by ultrafiltration. The generating CRP/sgRNA solution was adjusted to pH 7.5~8.0 with 10 mM NaHCO_3_, followed by addition of Cas9 to the solution under mild stirring at a Cas9/sgRNA molar ratio of 1: 1. The ^Cas9^CRP/sgRNA nanoparticle was obtained after incubation for 6 h. The nonresponsive ^Cas9^PP/sgRNA from PP polymer was prepared by similar steps. Free Cas9 was removed by ultrafiltration. The direct Cas9 RNP loading nanoparticle (CRP/RNP) was also prepared by vortex mixing of Cas9 RNP solution with CRP at a ratio of 1: 5 (v/v), followed by incubation for 6 h. Unbonded Cas9 RNP were removed by ultrafiltration. To determine the Cas9 mass in the nanoparticle, the ^Cas9^CRP/sgRNA nanoparticle was treated with H_2_O_2_ solution (10 mM) to release Cas9 and sgRNA, and the residual H_2_O_2_ was neutralized by ascorbic acid. Thereafter, the solution was subjected to BCA analysis for quantitative analysis of Cas9 content. The drug loading (DL%) and entrapment efficiency (EE%) of Cas9 were calculated as following Equation (1) and (2).

$\text{D}\text{L}\text{ }\text{(}\text{\%}\text{)}\text{ =}\frac{\text{mass of Cas9 in nanoparticle}}{\text{mass of nanoparticle}}\text{×100\%}$ (1)

$\text{EE}\text{ (}\text{\%}\text{)}\text{=}\frac{\text{mass of Cas9 in nanoparticle}}{\text{mass of Cas9 in feed}}\text{×100\%}$ (2)

The compartmentalized structure of ^Cas9^CRP/sgRNA nanoparticle was evaluated by scanning transmission electron microscopy (STEM, Talos F200S, Thermo Fisher Scientific Ltd., USA). The distribution of O, B, P, S celements was analyzed by energy dispersive X-ray spectrometer (EDS) mapping, where the O, B, P, S elements represented the whole nanoparticle, CRP polymer, sgRNA and Cas9 respectively. Moreover, the compartmentalized structure of ^Cas9^CRP/sgRNA nanoparticle was investigated by enzymic digestion. The ^Cas9^CRP/sgRNA nanoparticle was incubated with RNase A (10 μg/mL) and proteinase K (50 μg/mL) for 6 h. Thereafter, EDTA (10 mM) was added to the mixture, followed by heat at 95 ℃ for 5 min to inactivate the enzymes. CRP polymer in ^Cas9^CRP/sgRNA nanoparticle was degraded by H_2_O_2_ to release the payloads. 10% (w/v) sodium dodecyl sulfate (SDS) was added to prevent or dissociate the assembly between sgRNA and Cas9. The integrality of Cas9 and sgRNA was detected with SDS-PAGE and agarose gel electrophoresis. The direct Cas9 RNP loading nanoparticle of CRP/RNP, and naked Cas9 RNP were used as control.

**Preparation and characterization of ^Cas9^ARLP/sgRNA.** 50 mg of phosphatidylcholine (PC) was dissolved in 10 mL of mixed solvent (CH_3_OH: CHCl_3_ = 1: 1, v/v), and then the solvent was removed by rotary evaporation to form a lipid film. Next, the ^Cas9^CRP/sgRNA solution was used to dissolve the lipid film at proper weight ratio of ^Cas9^CRP/sgRNA to PC. After probe sonication (Beidi-900TE, Nanjing Beidi Experimental Instrument Co., Ltd., Jiangsu, China) at 200 W for 15 min in ice-water bath, the 4F-Angiopep-2 peptide was added to the ^Cas9^RLP/sgRNA emulsion, stirring for another 6 h to generate the ^Cas9^ARLP/sgRNA nanoparticle. The dynamic binding behavior between lipid-polymer hybrid nanoparticle and 4F-Angiopep-2 was detected by surface plasmon resonance (SPR). Briefly, the ^Cas9^RLP/sgRNA nanoparticle was fixed on LIP-1 sensor chip via hydrophobic interaction between lipid membrane and alkyl groups on sensor chip. The 4F-Angiopep-2 and 4F peptides were injected into the flow system, and the kinetic constants of binding were obtained using a 1: 1 Langmuir binding model via a BIA evaluation software.

The morphology of the nanoparticles was visualized by transmission electron microscopy (TEM, H-600, Hitachi, Ltd., Tokyo, Japan). The nanoparticles were adsorbed onto a copper grid, followed by phosphotungstic acid negative staining and drying with an infrared lamp for TEM analysis. The particle size, polydispersity index (PDI) and zeta potential of nanocomposites were measured by dynamic light scattering (DLS, Zetasizer, Malvern Panalytical Ltd., Malvern, UK), respectively. The integrity of sgRNA and CRP polymer during ^Cas9^ARLP/sgRNA nanoparticle preparation was further investigated. The sgRNA was released from ^Cas9^ARLP/sgRNA by incubation with 10 mM H_2_O_2_, and the samples were subjected to gel electrophoresis with untreated free sgRNA used as control. The CRP polymer was dissolved in water and sonicated. Thereafter, the sonicated CRP was subjected to thin-layer chromatography analysis, with untreated CRP polymer used as control.

**Design and screen of sgSTAT3.** The sgRNAs targeting STAT3 (sgSTAT3) were designed using a CRISPR design tool (http://www.e-crisp.org/). The sgSTAT3 and Cas9 were synchronously transfected into the GL261 cells with Lipofectamine™ CRISPRMAX™ reagent, where the concentration of Cas9 and sgRNA was 100 nM. The total RNA of treated GL261 cells was extracted by column extraction at 48 h post transfection, and the RNA concentration was measured by microspectrophotometer. The corresponding complementary DNA (cDNA) was prepared via reverse transcription with 1 μg RNA used as template. The STAT3 expression was determined by Real-time fluorescence quantification PCR (QuantStudio 3, Thermo Fisher Scientific).

***In vitro* drug release behavior.** The ^Cas9^ARLP/sgRNA and ^Cas9^ALP/sgRNA nanoparticles were dispersed in H_2_O_2_ solution of 0, 0.001, 0.1, 1 and 10 mM to analyze the release behavior of Cas9 and sgRNA. The mixtures were sampled at the predesigned time intervals and the H_2_O_2_ was eliminated by N-Acetyl-L-cysteine (NAC). The liberated Cas9 from the nanoparticles was separated by centrifugation and the Cas9 concentration was measured using BCA protein detection kit, and the sgRNA concentration was determined by fluorescence with Cy5-labeling. The sgRNA released from ^Cas9^ARLP/sgRNA nanoparticles was also detected by agarose gel electrophoresis analysis, with unresponsive ^Cas9^ALP/sgRNA nanoparticles used as control.

**Stability evaluation of ^Cas9^ARLP/sgRNA.** Stability in plasma. The freshly prepared ^Cas9^ARLP/sgRNA nanoparticle was dispersed in 10% plasma solution, followed by incubation at 37 ℃ for 24 h. The diameter and polydispersity index were measured at the specified time intervals. Storage stability. The freshly prepared ^Cas9^ARLP/sgRNA nanoparticle was stored in aseptic RNase-free water for 30 days. The ^Cas9^CRP/sgRNA nanoparticle was subjected to evaluation of stability in plasma at 37 °C and and storage stability at 4 °C. Samples were taken at the predesigned time intervals for monitoring the variations of particle size and polydispersity index.

**T7EI assay investigated the efficiency of insertion.** Genomic DNA of collected tissues or cells was extracted using the Genomic DNA Purification Kit following the manufacturer’s manual. The concentration of the proposed genomic DNA was determined by microspectrophotometer, and the fragment with cleavage site (about 800 bp) was amplified by PCR reaction for agarose gel electrophoresis. The PCR products were denatured at 95 °C for 10 min and reannealed at -2 °C per second temperature ramp to 85 °C, followed by a -1 °C per second ramp to 25 °C. The heterocomplexed PCR product (10 µL) was incubated with 5 U T7E1 enzyme at 37 °C for 15 min. The reaction was terminated by adding 0.25 M EDTA solution, and the cleaved amplicons were analyzed by 2% agarose gel electrophoresis with GelRed used to visualize the DNA.

**Cleavage activity of purified Cas9 and Cas9 released from the nanoparticles.** To evaluate the DNA cleavage activity of purified Cas9, the Cas9 was pre-incubated with sgEGFP to form the Cas9 RNP, followed by the addition of EGFP gene segment and reaction buffer. The mixture was incubated at 37 ℃ for 1 h and the cleavage product was analyzed by agarose gel electrophoresis to detect the Cas9 cleavage activity. The cleavage efficiency was calculated according to band intensities, using untreated EGFP band as control.

The DNA cleavage activity of Cas9 released from the nanoparticles. The nanoparticles were incubated in 10 mM H_2_O_2_ at 37 ℃ for 12 h to completely release the Cas9 and sgEGFP, and form the Cas9 RNP. The redundant H_2_O_2_ was removed with NAC, and the Cas9 cleavage activity could be estimated as described above.

***In vitro* biosafety evaluation of the compartmentalized nanoparticles.** The GL261 cells were plated at a density of 1 × 10^4^ cells in 96-well plates. After overnight culture, cells were treated with ^Cas9^ARLP/sgNC, ^Cas9^ALP/sgNC, and ^Cas9^RLP/sgNC at Cas9 concentrations of 0.005, 0.01, 0.02, 0.03, 0.05, 0.08 and 0.1 μg μL^-1^. After incubation for 48 h, cell proliferation was quantified using the standard dimethyl thiazolyl diphenyl tetrazolium salt (MTT) assay. Briefly, 10 mg mL^-1^ MTT in PBS was added to the cells, resulting in a final MTT concentration of 1 mg mL^-1^. The cells were cultured for additional 4 h. Afterward, the media were removed and 150 μL DMSO was added to each well to dissolve the formazen crystals. The absorbance was measured by a microplate reader (Varioskan LUX, Thermo Fisher Scientific, Inc., MA, USA) at 570 nm. The following Equation (3) calculated the viability of cells:

$\text{Cell viabilit}\text{y (}\text{\%}\text{)}\text{=}\frac{\text{O.D.}_{\text{Sample}}}{\text{O.D.}_{\text{Control}}}\text{×100\%}$ (3)

**Intracellular co-delivery of Cas9 and sgRNA.** To evaluate cellular uptake of ^Cas9^ARLP/sgRNA nanoparticle, the FAM fluorophore was conjugated on PEI polymer via amidation, and the generated FAM-PEI was subjected to the synthesis of CRP polymer to yield the FAM-CRP polymer. FAM-CRP polymer was used to fabricate the FAM-labeled nanoparticles for incubation with GL261 cells. At predesigned time intervals, the cells were fixed and stained with DAPI, followed by fluorescence imaging. Simultaneously, cells treated with FAM-labeled nanoparticles were collected for flow cytometry analysis on fluorescence intensity.

To track nanocomposites in the GL261 cells, Cas9 was labeled with Cy3 and sgRNA was labeled with Cy5 to prepare the dual-labeled ^Cas9^ARLP/sgRNA, ^Cas9^RLP/sgRNA and ^Cas9^ALP/sgRNA (Cas9 200 nM). The Cy3-labeled Cas9 (Cy3-Cas9) was obtained through amide reaction. In brief, the Cy3-NHS and Cas9 solution were mixed at 50: 1 (n/n) and stirred slowly for 8 h. The Cy3-labeled Cas9 was purified by dialysis (MWCO: 3500 Da) for 12 h, followed by ultrafiltration centrifuge to collect the Cy3-Cas9. To prepare the Cy5-labeled sgRNA (Cy5-sgRNA), the Cy5-modified RNA probe and sgRNA were added to the RNA annealing buffer at a molar ratio of 1: 1 and annealed on a PCR instrument to yield the Cy5-sgRNA. The annealing procedure is 30 ℃, 30 min, 95 ℃, 5 min, cooling to 25 ℃. The GL261 cells (2 × 10^5^ cells well^-1^) were cultured in glass-bottom dishes for 8 h with the dual-labeled nanoparticles, respectively. Thereafter, the cells were observed under a confocal microscope (CLSM, LSM700, Carl Zeiss AG, Oberkochen, Germany) and analyzed with a flow cytometer (FACS Celesta, Becton, Dickinson and Company, NJ, USA).

***In vitro* blood-brain barrier model and penetration capacity.** The hCMEC/D3 cells were cultured on the apical chamber of the transwell insert (0.4 μm chambers, Corning Co, USA) at a density of 1 × 10^4^ cells well^-1^, with the cell-free chamber used as control. The culture medium was replaced every 48 h. The trans-endothelial electrical resistance (TEER) values were measured every 24 h by a cell resistance meter. When the TEER value reached 100-120 Ω/cm^2^ and could remain stable, the *in vitro* BBB model was successfully constructed.

To examine BBB permeability of different preparations, the FAM-CRP polymer and Cy3-Cas9 were employed to fabricate the dual-labeled nanoparticles. The medium in the upper chamber was replaced with the medium containing dual-labeled nanoparticles, and fresh medium was added to the lower chamber. After incubation for 4 h, the fluorescence intensity of dual-labeled nanoparticles in the lower chamber was determinated using a microplate reader to calculate the BBB penetration efficiency. Furthermore, the dual-labeled nanoparticles collected from the lower chamber were used to incubate with GL261 cells for cellular uptake evaluation by fluorescence imaging.

**Gene editing efficiency.** The STAT3 gene editing efficiency was investigated at the levels of genome, mRNA and protein. The GL261 cells were placed onto 6-well plates at a density of 1 × 10^5^ cells well^-1^. After culture for 12 h, the cells were transfected with different formulations at the Cas9 concentration of 200 nM, and incubated for 48 h. The genome DNA was extracted from the treated GL261 cells via column extraction. The STAT3 sequence was amplified by PCR and subjected to T7EI assay to detect the indels. Total RNA of each group was extracted by column extraction and the RNA concentration was measured by microspectrophotometer. The STAT3 transcription level was determined by Real-time fluorescence quantification PCR. Furthermore, the cells were lysed in PMSF containing RIPA lysis buffer in ice bath. The total protein concentrations were determined by BCA assay. After being separated through a 10% SDS-PAGE, the protein was transferred onto the poly vinylidene fluoride (PVDF) membrane. Then, the membranes were blocked with 5% BSA for 2 h at room temperature, followed by incubating with STAT3 primary antibodies at 4 ℃ overnight. After being washed with TBST buffer, the membranes were incubated with horseradish peroxidase (HRP)-linked secondary antibodies (goat anti-rabbit IgG H&L, rabbit anti-mouse IgG H&L, 1: 10000, Servicebio) for 1 h at room temperature. The membranes were stained with enhanced chemiluminescence (ECL) for imaging with a gel imaging system (Tanon 5200, Tanon Technology Co., Ltd., Shanghai, China).

The variations of STAT3 downstream factors after treatment with different formulations were also investigated. The total RNA of cells was extracted by column extraction for quantitative real-time PCR analysis on VEGF, IL-6 and IL-10 expression. The supernatant of GL261 cells in each group was collected to detect the concentration of VEGF, IL-6 and IL-10 by enzyme-linked immunosorbent assay (ELISA). The supernatant of GL261 cells in each group was also collected to evaluate the immunosuppressive microenvironment reversion upon treatment with different formulations. The BV-2 microglial cells were induced with IL-4 (20 ng μL^-1^) to get the BV-2 (M2) microglial cells. The BV-2 (M2) microglial cells were cultured in the collected supernatant of GL261 cells for 48 h. Thereafter, the microglial cells were harvested and stained with anti-CD206-APC and anti-CD80-PE, and then subjected to flow cytometry analysis on M1/M2 polarization.

**STAT3 knockout sequencing validation and off-target analysis.** The GL261 cells were transfected with ^Cas9^ARLP/sgSTAT3 and ^Cas9^ARLP/sgNC and cultured for 48 h. The genomic DNA was extracted and the target gene fragment containing the targeting site was amplified by PCR. The 4 μL amplified gene segment was mixed with 1 μL 5 × TA/Blunt-Zero Cloning Mix to construct the TOPO cloning reaction system, followed by reaction for 5 min at 37 ℃ and setting aside on ice after the reaction. The ligated products were transferred into Escherichia coli and screened with kanamycin and ampicillin at the same time. Monoclonal bacteria with different STAT3 fragments were isolated and the plasmids were extracted and sequenced. The Sanger sequencing data were analyzed by Applied Biosystems™ SeqScreener. The potential off-target sites for sgSTAT3 were predicted using Cas-OFFinder (http://www.rgenome.net/cas-offinder/). The sequences of top three off-target sites were amplified by PCR using genome DNA as template respectively, and the off-target editing was detected by T7EI assay.

**GBM-bearing mice and intracranial needle implantation.** The mice were anesthetized and positioned in the Kopf Stereotaxic Alignment System for inoculation with 1 × 10^5^ cells using automatic syringe pump. Injections were made to the right frontal lobe, ~1.8 mm lateral, 2 mm caudal from bregma, and at a depth of 2.3 mm. Bioluminescence imaging (VISQUE, Vieworks Co., Ltd., Gyeonggi-do, Korea) was performed every 5 days after tumour cell implantation to monitor tumour growth. The D-luciferin sodium salt was injected at 15 mg kg^-1^ subcutaneously, and the imaging was conducted at 15 min post the injection. Bioluminescence analysis was conducted using the living image software.

To implant an intracranial needle, skull of the mice was exposed, and four small screws were fixed around the administration position. Thereafter, the long guide tube of 5 mm was inserted into the 3 mm under the skull. The cannula and screws were fixed in place with self-curing acrylic dental cement and inserted into the cannula for head suture. The recovery time of the operation was about 1 week.

***In vivo* glioblastoma targeting.** The BBB penetration of nanoparticles was evaluated by the imaging system (VISQUE, Vieworks Co., Ltd., Gyeonggi-do, Korea). GBM-bearing mice were intravenously injected with Cy5-labeled formulations (1.5 mg kg^-1^ of Cy5-sgRNA), respectively (n=3). The fluorescence images of mice were taken at 2, 4, 6, 10, 24 and 48 h. Thereafter, the mice were sacrificed, and the brain and major organs were isolated for *ex vivo* imaging. The brains of each group were collected and lysed for quantitative analysis on intracranial nanoparticle distribution, where the nanoparticle content was represented by sgRNA concentration in brain tissues. Moreover, intratumoural distribution of nanoparticles was inspected by fluorescence imaging of frozen section.

**Glioblastoma progression monitoring with MRI and bioluminescence imaging.** The GBM-bearing mice were divided to six groups randomly: (i) ^Cas9^ARLP/sgSTAT3; (ii) ^Cas9^RLP/sgSTAT3; (iii) ^Cas9^ALP/sgSTAT3; (iv) ^Cas9^ARLP/sgNC; (v) ^Cas9^ARLP/sgSTAT3 + VEGF; (vi) saline (Cas9 at 100 mg kg^-1^ and VEGF at 3 ng kg^-1^). After the tumour-bearing mice were anesthetized by intraperitoneal injection of chloral hydrate, 100 μL gadolinium diethylene-trianmine pentaacetic acid (Gd-DTPA) was injected intraperitoneally 1 min before scanning. And then the mice were fixed in the animal coil in prone position to start MRI scanning. The scanning parameters are as follows: TR = 500 ms, TE = 10.67 ms, Fov = 100 mm, FA = 152.9°, slice thickness = 1 mm, number of layers = 10, matrix = 256 × 256. The glioblastoma progression in each group was monitored by the MRI scanning during the treatment. Moreover, the mice were subjected to bioluminescence imaging to evaluate the glioblastoma progression. The D-Luciferin was administrated via intraperitoneal injection at a dosage of 15 mg/kg and the images were taken at 15 min post administration. During the *in vivo* anti-glioblastoma investigation, the mice were euthanized when they became moribund, i.e. rapid loss of weight, impaired ambulation which prevents animals from reaching food or water, lack of physical or mental alertness, difficult labored breathing, and inability to remain upright, according to NIH guidelines (*Institutional Animal Care and Use Committee Guidebook*) and the survival time was recorded.

***In vivo* gene editing efficiency.** After MRI scanning and bioluminescence imaging for the last time, the mice were euthanatized and the glioblastoma tissue in each group was collected for T7E1, qPCR and western blot analysis. The genome DNA was extracted from the glioblastoma tissue via column extraction. The STAT3 sequence was amplified by PCR and subjected to T7EI assay to detect the indels. Total RNA of each group was extracted by column extraction and the STAT3 transcription level was determined by Real-time fluorescence quantification PCR. The total protein of glioblastoma tissue was extracted by RIPA lysis buffer. The samples were analyzed by western blot to detect the STAT3 protein level.

***In vivo* vessel normalization and immune reprogramming assays.** Three mice of each group were sacrificed at 15 d, 24 d and 30 d, and brain tissues were dissected and processed for immunofluorescence (IF) staining with tumour endothelial cells marker (CD31), vascular cell adhesion molecule-1 (VCAM-1) and intercellular adhesion molecule-1 (ICAM-1) to investigate the tumour vessels according to the manufacturer’s instructions. Slides were observed with confocal fluorescence microscopy (ECLIPSE TI, Nikon, Japan). The glioblastoma tissue in brain was separated and used to prepare homogenate. The IL-6, IL-10 and VEGF in glioblastoma homogenate were detected by ELISA.

On the third day after the last administration, three mice in each group were euthanatized and the brains were collected to separate the glioma tissue for cell suspension preparation. The cell suspension was divided into four, and to detect the different immune cells via flow cytometry. The biomarkers for cytotoxic T lymphocytes (CTLs) were CD3+, CD4−, CD8+; The biomarkers for T helper cells (Th) were CD3+, CD4+, CD8−; The biomarkers for effector T cells (T_effs_) were CD4+, CD25+, FoxP3−; The biomarkers for regulatory T cells (T_regs_) were CD4+, CD25+, FoxP3+; The biomarkers for M1 macrophages were F4/80+, CD80+, CD206−; The biomarkers for M2 macrophages were F4/80+, CD80−, CD206+; The biomarkers for mature dendritic cells (DCs) were CD11c+, CD80+, CD86+; The biomarker for all DCs was CD11c+.

**Safety profiles evaluation.** To evaluate the safety of nanocomposites, the nanoparticle-induced hemolysis was investigated. The fresh rabbit red blood cells (RBC) were collected by centrifugation at 1500 rpm for 10 min. The ^Cas9^ARLP/sgRNA, ^Cas9^RLP/sgRNA and ^Cas9^ALP/sgRNA nanoparticles were diluted with saline at various concentrations and mixed with 2% RBCs suspension at volume ratio of 1: 1. The distilled water and saline mixed with 2% RBCs were used as positive (A_100%_) and negative (A_0%_) controls, respectively. All the samples were incubated at 37 ℃ for 1 h. Thereafter, the supernatant was collected by centrifugation at 3000 rpm for 10 min and detected by spectrophotometry at 540 nm. The percentage of hemolysis was calculated as Equation (4).

$\text{Hemolysis (\%)=}\frac{\text{A}_{\text{Sample}}\text{-}\text{A}_{\text{0\%}}}{\text{A}_{\text{100\%}}\text{-}\text{A}_{\text{0\%}}}\text{×100\%}$ (4)

For the biosafety analysis, the mice were treated with saline, ^Cas9^ARLP/sgSTAT3, ^Cas9^ALP/sgSTAT3, ^Cas9^RLP/sgSTAT3 (Cas9 at 100 mg kg^-1^ and VEGF at 3 ng kg^-1^). After administration for 5 times, the mice were sacrificed and the major organs were collected for HE staining. Organs from the saline-treated mice were used as control. And the serum samples were collected for the test of alanine aminotransferase (ALT), alkaline phosphatase (ALP), blood ureanitrogen (BUN) and creatinine (CRE).

**Statistical analysis.** All the details about sample size, data presentation, statistical analysis and significant differences are provided in the figure captions. Unpaired two-tailed student’s t-test was used for two-group comparison. One-way ANOVA, followed by Tukey post hoc analysis was used for multi-group comparison. The differences were displayed as significant for *p < 0.05, **p < 0.01 and ***p < 0.001. Data were expressed as mean ± standard deviation (SD). Statistical analysis of data was performed by Prism GraphPad 9 software.

**Supporting references**

[1] a) J. F. Berry, E. Bill, E. Bothe, T. Weyhermuller, K. Wieghardt, *J Am Chem Soc* **2005**, 127, 11550. b) H. Cheng, Z. Jiang, C. Sun, Z. Wang, G. Han, X. Chen, T. Li, Z. Fan, F. Zhang, X. Yang, L. Lv, H. Zhang, J. Zhou, Y. Ding, *Chem Eng J* **2022**, 427: 131672.

Figure S1.


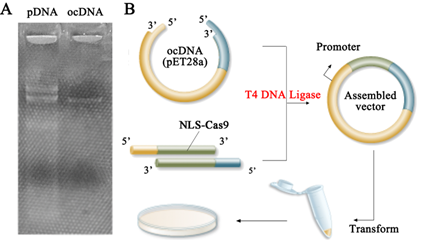


**Figure S1.** (A) Double enzyme digestion verification performed by agarose gel electrophoresis experiment. (B) Schematic of plasmid construction via ligation reaction.

Figure. S2.


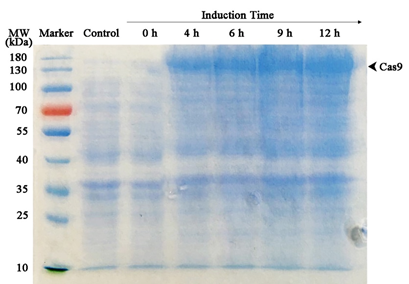


**Figure S2.** SDS-PAGE of Cas9 prokaryotic expression after inducted by 0.1 mM IPTG at 16 ℃ for different durations. BL21(DE3) cultured at 16 ℃ without IPTG for 12 h was used as control.

Figure. S3.


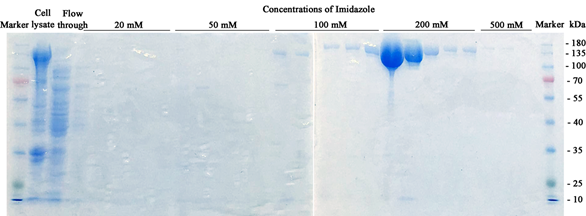


**Figure S3.** The SDS-PAGE electrophoresis results of Cas9 purified conditions screening, which principium is the affinity chromatography of His-tag and Ni column. The Cas9 band should be located between 135 kDa and 180 kDa.

Figure. S4.


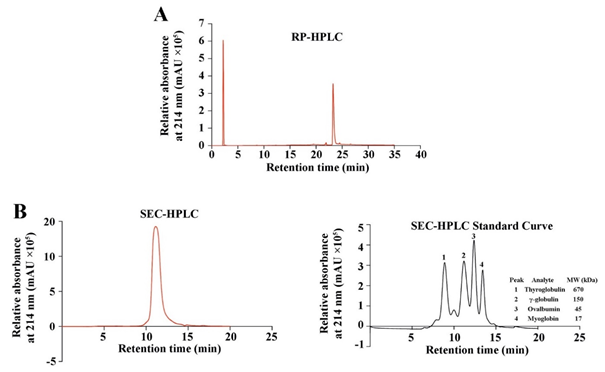


**Figure S4.** The purity of Cas9 was analyzed by (A) RP-HPLC and (B) SEC-HPLC. The thyroglobulin, γ-globulin, ovalbumin, and myoglobin were used as standard proteins.

Figure. S5.


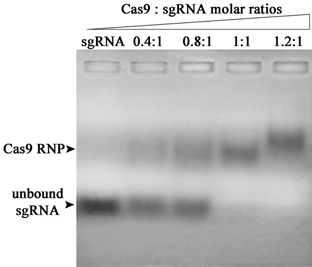


**Figure S5.** The binding ability of purified Cas9 protein and sgRNA after co-incubated under 37 ℃ for 10 min.

Figure. S6.


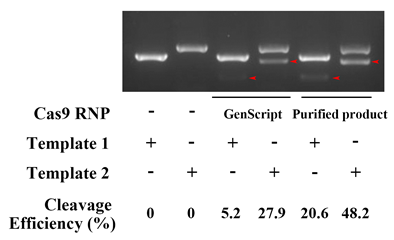


**Figure S6.** The gene cleavage capability of purified Cas9 compared with commercialized Cas9 (GenScript). The cleaved bands were indicated with red arrows.

Figure. S7.


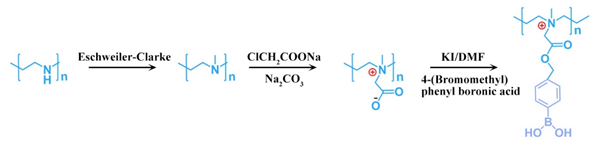


**Figure S7.** The synthetic route of the CRP polymer.

Figure. S8.


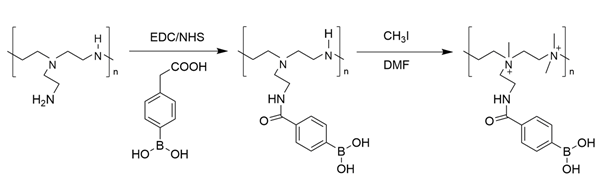


**Figure S8.** The synthetic route of the PP polymer.

Figure. S9.


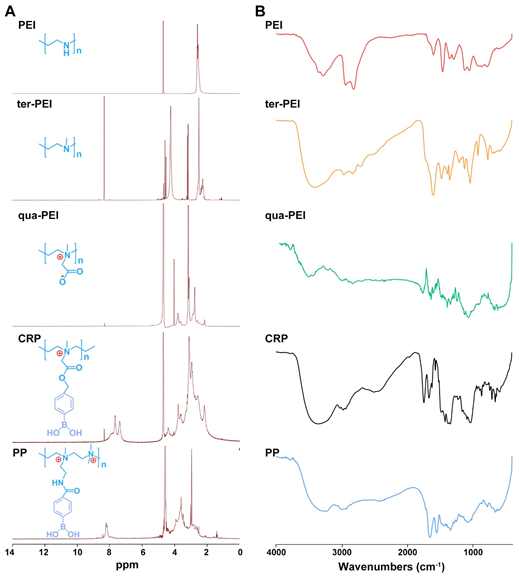


**Figure S9.** (A) ^1^H-NMR spectra of PEI, ter-PEI, qua-PEI, CRP and PP polymer. The polymers were dissolved in D_2_O at a concentration of 10 mg/mL. (B) FTIR spectra of PEI, ter-PEI, qua-PEI, CRP and PP polymer.

Figure. S10.


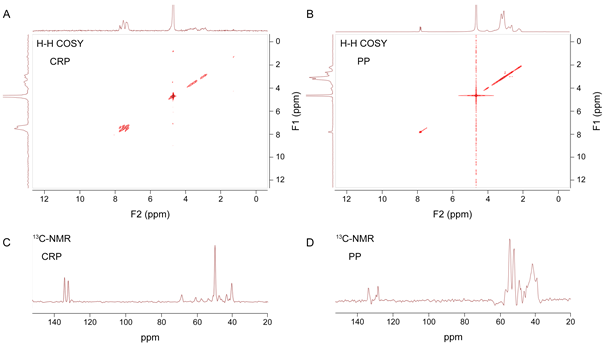


**Figure S10.** (A) H-H COSY spectra of CRP polymer. (B) H-H COSY spectra of PP polymer. (C) ^13^C-NMR of CRP polymer. (D) ^13^C-NMR of PP polymer.

Figure. S11.


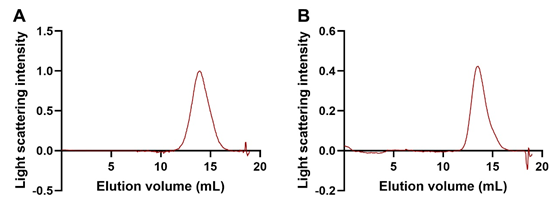


**Figure S11.** SEC-elution patterns of CRP (A) and PP (B) polymers dissolved in DMF.

Figure. S12.


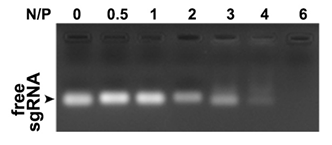


**Figure S12.** Agarose gel electrophoresis analysis of CRP/sgRNA polyplexes at different N/P molar ratios.

Figure. S13.


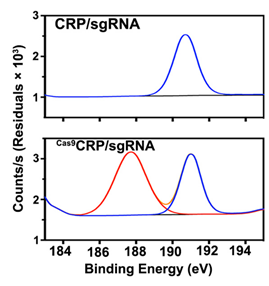


**Figure S13.** The binding energy of boron in CRP/sgRNA and ^Cas9^CRP/sgRNA measured by XPS.

Figure. S14.


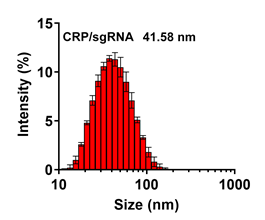


**Figure S14.** Diameter of CRP/sgRNA nanocomplex.

Figure. S15.


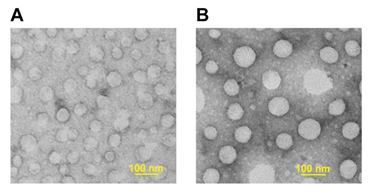


**Figure S15.** TEM overview images of ^Cas9^CRP/sgRNA (A) and ^Cas9^ARLP/sgRNA (B) nanoparticles.

Figure. S16.


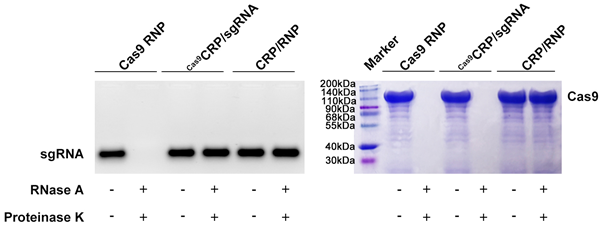


**Figure S16.** The agarose gel electrophoresis and SDS-PAGE of Cas9 RNP, ^Cas9^CRP/sgRNA and CRP/RNP after treatment with RNase A and proteinase K.

Figure. S17.


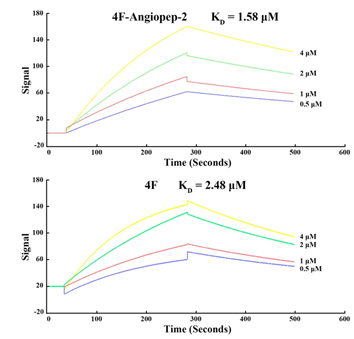


**Figure S17.** The binding affinity of 4F-Angiopep-2 and 4F peptides to ^Cas9^RLP/sgRNA measured by SPR. The kinetic constants of binding were obtained using 1:1 Langmuir binding model via a TraceDrawer software (n =4).

Figure. S18.


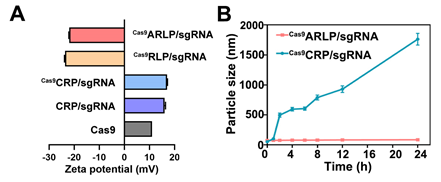


**Figure S18.** (A) The zeta potential of Cas9, CRP/sgRNA, ^Cas9^CRP/sgRNA, ^Cas9^RLP/sgRNA, and ^Cas9^ARLP/sgRNA. (B) The biostability of ^Cas9^CRP/sgRNA and ^Cas9^ARLP/sgRNA nanoparticle in serum. Data are given as means ± S.D., n=3.

Figure. S19.


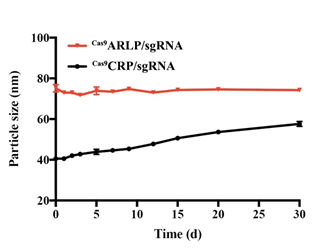

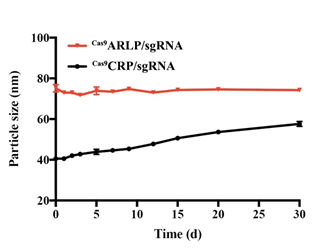


**Figure S19.** The stability of ^Cas9^ARLP/sgRNA, with ^Cas9^CRP/sgRNA used as control. ^Cas9^ARLP/sgRNA and ^Cas9^CRP/sgRNA were stored at 4 ℃ in saline for 30 d. The particle sizes were measured every few days. Data are given as means ± S.D., n = 3.

Figure. S20.


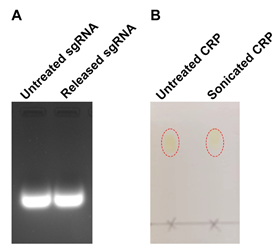


**Figure S20.** (A) Agarose gel electrophoresis of untreated sgRNA and sgRNA released from ^Cas9^ARLP/sgRNA nanoparticle. (B) Thin layer chromatography analysis of untreated CRP polymer and sonicated CRP polymer with CRP visualized by iodine staining.

Figure. S21.


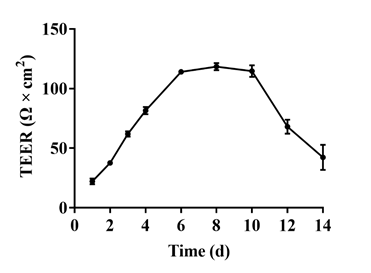


**Figure S21.** The TEER value of the hCMEC/D3 cell monolayer in 14 days. Data are given as means ± S.D., n = 6.

Figure. S22.


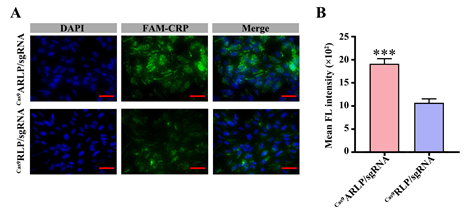


**Figure S22.** (A) Cellular uptake of ^Cas9^ARLP/sgRNA and ^Cas9^RLP/sgRNA, with the CRP polymer modified with FAM (FAM-CRP). The fluorescence images obtained by inverted fluorescence microscope. (B) Average fluorescence intensity of GL261 cells detected by flow cytometry. Data are given as means ± S.D., n = 3, ***p < 0.001.

Figure. S23.


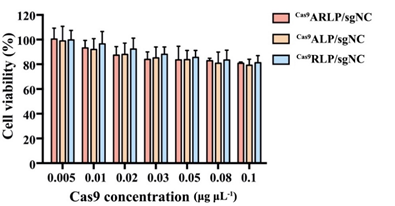


**Figure S23.** The cytotoxicity of ^Cas9^ARLP/sgNC, ^Cas9^ALP/sgNC, and ^Cas9^RLP/sgNC mearsured by MTT assay. Data are given as means ± S.D., *n* = 6.

Figure. S24.


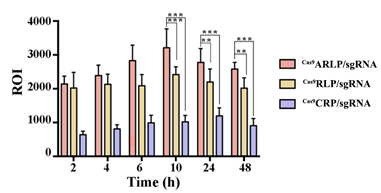


**Figure S24.** The quantitative statistics of the *in vivo* biodistribution in glioma region. Data are given as means ± S.D., n = 3, **p < 0.01, ***p < 0.001.

Figure. S25.


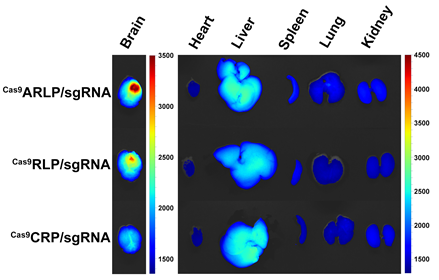


**Figure S25.** *Ex vivo* imaging of brains and main organs upon Cy5 channel.

Figure. S26.


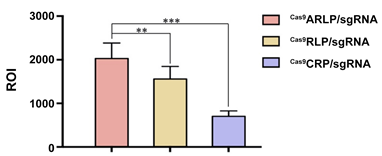


**Figure S26.** The quantitative statistics of the *ex vivo* biodistribution in glioma region. Data are given as means ± S.D., n = 3, **p < 0.01, ***p < 0.001.

Figure. S27.


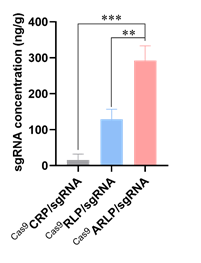


**Figure S27.** Quantitative intracerebral distribution of nanopartciles at 48 h post administration. The content of nanoparticles was represented by sgRNA concentrations in tissues. Data are given as means ± S.D., n = 3, **p < 0.01, ***p < 0.001.

Figure. S28.


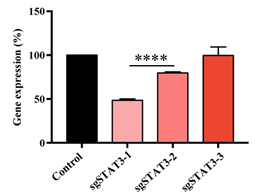


**Figure S28.** The knockout effect screening measured by quantitative polymerase chain reaction. Data are given as means ± S.D., n = 3, ****p < 0.0001.

Figure. S29.


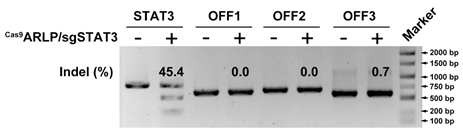


**Figure S29.** The off-target indel efficiency of ^Cas9^ARLP/sgSTAT3 in GL261 cells determined by T7EI assay.

Figure. S30.


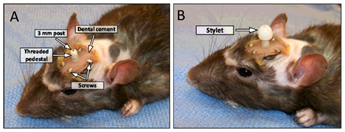


**Figure S30.** The scheme of intracranial needle implantation.

Figure. S31.


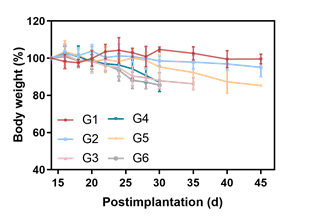


**Figure S31.** The body weight changes of mice in different groups within 45 days. G1: ^Cas9^ARLP/sgSTAT3, G2: ^Cas9^RLP/sgSTAT3, G3: ^Cas9^ALP/sgSTAT3, G4: ^Cas9^ARLP/sgNC, G5: ^Cas9^ARLP/sgSTAT3+VEGF, G6: saline. Data are given as means ± S.D., n = 6, ***p < 0.001.

Figure. S32.


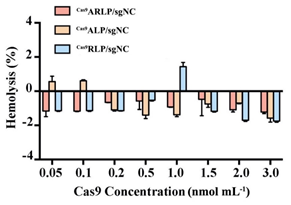


**Figure S32.** Hemolysis percentage of ^Cas9^ARLP/sgNC, ^Cas9^ALP/sgNC, and ^Cas9^RLP/sgNC, at different concentrations. Data are given as means ± S.D., n = 3.

Figure. S33.


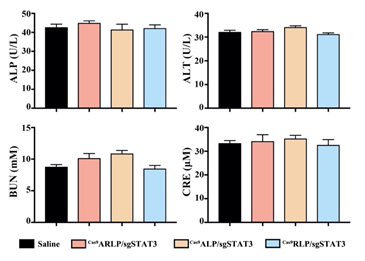


**Figure S33.** Evaluation of liver functions (ALP and ALT) and renal functions (BUN and CRE) of tumour-bearing mice after treatment of different preparations. Data are given as means ± S.D., n = 3.

Figure. S34.


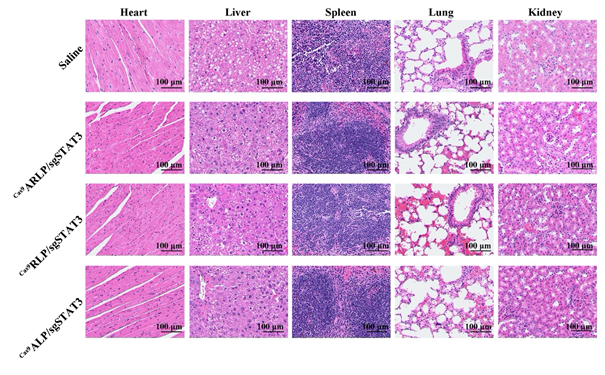


**Figure S34.** Representative HE staining images of heart, liver, spleen, lung and kidney sections after different treatments.

Table S1.

**Table S1.** Characterization of Cas9 and sgRNA loaded nanoparticles. Data are given as means ± S.D., n = 3.

| Loading form | Encapsulation efficiency of Cas9 (%) | Drug loading of Cas9 (%) | Diameter (nm) | polydispersity index |
| --- | --- | --- | --- | --- |
| Compartmentalized loading of Cas9 and sgRNA | 91.46±5.26 | 16.93±2.78 | 47.99±1.94 | 0.161±0.023 |
| Direct loading of Cas9 RNP | 53.97±8.83 | 5.19±1.94 | 625.68 ± 98.53 | 0.419 ± 0.032 |

Table S2.

**Table S2.** Raw data of diameter distribution of ^Cas9^CRP/sgRNA and ^Cas9^ARLP/sgRNA, n = 3.

| ^Cas9^CRP/sgRNA | | | ^Cas9^ARLP/sgRNA | | |
| --- | --- | --- | --- | --- | --- |
| Size | Intensity (%) | SD | Size | Intensity (%) | SD |
| 18.17 | 0.2 | 0.2 | 21.04 | 0.1 | 0.1 |
| 21.04 | 1.2 | 0.3 | 24.36 | 1.1 | 0.4 |
| 24.36 | 3.2 | 0.3 | 28.21 | 3.2 | 0.7 |
| 28.21 | 5.8 | 0.3 | 32.67 | 6 | 1.1 |
| 32.67 | 8.5 | 0.5 | 37.84 | 8.9 | 1.2 |
| 37.84 | 10.8 | 0.8 | 43.82 | 11.3 | 1 |
| 43.82 | 12.3 | 0.8 | 50.75 | 12.8 | 0.6 |
| 50.75 | 12.7 | 0.7 | 58.77 | 13.2 | 0.4 |
| 58.77 | 12.1 | 0.4 | 68.06 | 12.5 | 0.7 |
| 68.06 | 10.6 | 0.2 | 78.82 | 10.8 | 1 |
| 78.82 | 8.5 | 0.6 | 91.28 | 8.4 | 1.2 |
| 91.28 | 6.2 | 0.9 | 105.7 | 5.8 | 1.2 |
| 105.7 | 3.9 | 1 | 122.4 | 3.4 | 0.9 |
| 122.4 | 2.1 | 0.9 | 141.8 | 1.5 | 0.6 |
| 141.8 | 0.9 | 0.6 | 164.2 | 0.4 | 0.2 |
| 164.2 | 0.2 | 0.2 |  |  |  |

Table S3.

**Table S3.** The sgRNA targeting sequences.

| Samples | Sequences (5’→3’) |
| --- | --- |
| sgSTAT3-1 | GGTAGCGTGTGTCCAGCTGC |
| sgSTAT3-2 | TGGGGAAGCTGTCGCTGTAC |
| sgSTAT3-3 | GGAACTGCCGCAGCTCCATG |

Table S4.

**Table S4** The sequences of the qPCR primers.

| Samples | Sequences (5’→3’) |
| --- | --- |
| STAT3-F | GCTAGCCTGAGACCCTGTTT |
| STAT3-R | TAGCACACGGGCCTGAATAC |
| VEGF-F | AACCTCCTCAAACCGTTGGC |
| VEGF-R | GCAGCGACAAGGCAGACTAT |
| IL-6-F | GTGACTCCAGCTTATCTCTTGGT |
| IL-6-R | CAACGATGATGCACTTGCAGA |
| IL-10-F | CTGCTGATCCTCATGCCAGT |
| IL-10-R | GCTCTTGCACTACCAAAGCC |
| GAPDH-F | CAGGTTTCCCATCCCCACATA |
| GAPDH-R | GGTTGTCTCCTGCGACTTCA |

Table S5.

**Table S5.** Potential off-target sites when exists 4 maximum mismatches.

| Sites | Genomic hit | Number of mismatches |
| --- | --- | --- |
| STAT3 | GCAGCTGGACACACGCTACC-TGG | 0 |
| OFF1 | GTAGCTGGAAGCACGCTACC-AGG | 3 |
| OFF2 | CCAGCTGGGCTCTCGCTACC-CGG | 4 |
| OFF3 | GGAGCAGTACACGCGCTACC-AGG | 4 |
